# High‐Speed Hyperspectral Imaging for Near Infrared Fluorescence and Environmental Monitoring

**DOI:** 10.1002/advs.202415238

**Published:** 2025-03-04

**Authors:** Jan Stegemann, Franziska Gröniger, Krisztian Neutsch, Han Li, Benjamin Scott Flavel, Justus Tom Metternich, Luise Erpenbeck, Poul Bering Petersen, Per Niklas Hedde, Sebastian Kruss

**Affiliations:** ^1^ Department of Chemistry and Biochemistry Ruhr University Bochum 44801 Bochum Germany; ^2^ Fraunhofer Institute for Microelectronic Circuits and Systems 47057 Duisburg Germany; ^3^ Department of Mechanical and Materials Engineering University of Turku Turku FI‐20014 Finland; ^4^ Turku Collegium for Science Medicine and Technology University of Turku Turku FI‐20520 Finland; ^5^ Institute of Nanotechnology Karlsruhe Institute of Technology 76021 Karlsruhe Germany; ^6^ Department of Dermatology University Hospital Münster 48149 Münster Germany; ^7^ Beckman Laser Institute & Medical Clinic University of California Irvine CA 92612 USA

**Keywords:** fluorescence, hyperspectral imaging, label‐free, near infrared, spectral phasor

## Abstract

Hyperspectral imaging captures both spectral and spatial information from a sample but is intrinsically slow. The near infrared (NIR, > 800 nm) is advantageous for imaging applications because it falls into the tissue transparency window and also contains vibrational overtone and combination modes useful for molecular fingerprinting. Here, fast hyperspectral NIR imaging is demonstrated using a spectral phasor transformation (HyperNIR). A liquid crystal variable retarder (LCVR) is used for tunable, wavelength‐dependent sine‐ and cosine‐filtering that transforms optical signals into a 2D spectral (phasor) space. Spectral information is thus obtained with just three images. The LCVR can be adjusted to cover a spectral range from 900 to 1600 nm in windows tunable from 50 to 700 nm, which enables distinguishing NIR fluorophores with emission peaks less than 5 nm apart. Furthermore, label‐free hyperspectral NIR reflectance imaging is demonstrated to identify plastic polymers and monitor in vivo plant health. The approach uses the full camera resolution and reaches hyperspectral frame rates of 0.2 s^−1^, limited only by the switching rate of the LCVR. HyperNIR facilitates straightforward hyperspectral imaging for applications in biomedicine and environmental monitoring.

## Graphical abstract

This study introduces a fast hyperspectral imaging method in the near‐infrared range (HyperNIR). Combining tunable polarization optics with a phasor‐based approach, HyperNIR captures detailed spectral information with just three images. The applicability is showcased by distinguishing NIR fluorophores that are separated by 5 nm. Furthermore, plastic polymers are identified and plant health is monitored, highlighting its potential for environmental applications.

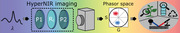

## 1 Introduction

Label‐free imaging techniques enable nondestructive and high‐resolution interrogation of materials and biomedical samples without the need for exogenous labels.^[^ ^1^ ^]^ These techniques leverage inherent optical properties of samples to generate contrast and extract detailed information about their structure, composition, and function. Prominent label‐free imaging modalities include phase contrast,^[^ ^2^, ^3^ ^]^ polarization,^[^ ^4^ ^]^ infrared‐absorption,^[^ ^5^ ^]^ autofluorescence,^[^ ^6^ ^]^ photoacoustic,^[^ ^7^, ^8^ ^]^ scattering‐based,^[^ ^9^, ^10^, ^11^ ^]^ or Raman^[^ ^1^ ^]^ microscopy. Such techniques are employed not only for basic research but also for clinical diagnostics^[^ ^1^ ^]^ and drug development.^[^ ^12^ ^]^ In materials science, these techniques contribute to the characterization of materials at the micro‐ and nano‐scale, aiding in the design and optimization of novel materials for diverse applications.^[^ ^13^ ^]^ Despite many advances, weak signals, limited penetration depth, and specialized instrumentation limit the use of these techniques.^[^ ^1^ ^]^

The near‐infrared (NIR) range of the electromagnetic spectrum (800–2500 nm) corresponds to overtone and combinational vibrational modes. For instance, O─H, N─H, and C─H bonds exhibit characteristic absorption features in the NIR region. NIR spectroscopy exploits these absorption features to characterize samples rapidly and nondestructively.^[^ ^14^ ^]^ Reflected or transmitted NIR light from a material provides therefore information about chemical composition, structure, and physical properties. This analytical technique finds widespread use in pharmaceutical,^[^ ^15^ ^]^ agriculture,^[^ ^16^, ^17^ ^]^ and food^[^ ^18^ ^]^ industries, as well as in biomedical research.^[^ ^19^, ^20^ ^]^ The NIR is also highly beneficial for biomedical applications due to the so‐called “optical windows” from 650 to 1350 nm and above 1450 nm.^[^ ^21^ ^]^ As absorption and scattering are reduced in this region, light can penetrate deeper into biological tissue than visible (Vis) and mid‐IR light. Ongoing developments in instrumentation and data analysis methods (chemometrics) enhance the capabilities and applicability of NIR spectroscopy.^[^ ^14^ ^]^ This progress is complemented by the development of novel NIR fluorescent nanomaterials.^[^ ^22^ ^]^ They enable NIR fluorescence imaging with high signal‐to‐noise ratios. One example are single‐walled carbon nanotubes (SWCNTs) that can be chemically tailored as biosensors for detection and imaging of signaling molecules, pathogens, or disease markers.^[^ ^23^, ^24^, ^25^, ^26^ ^]^

**Figure 1:**
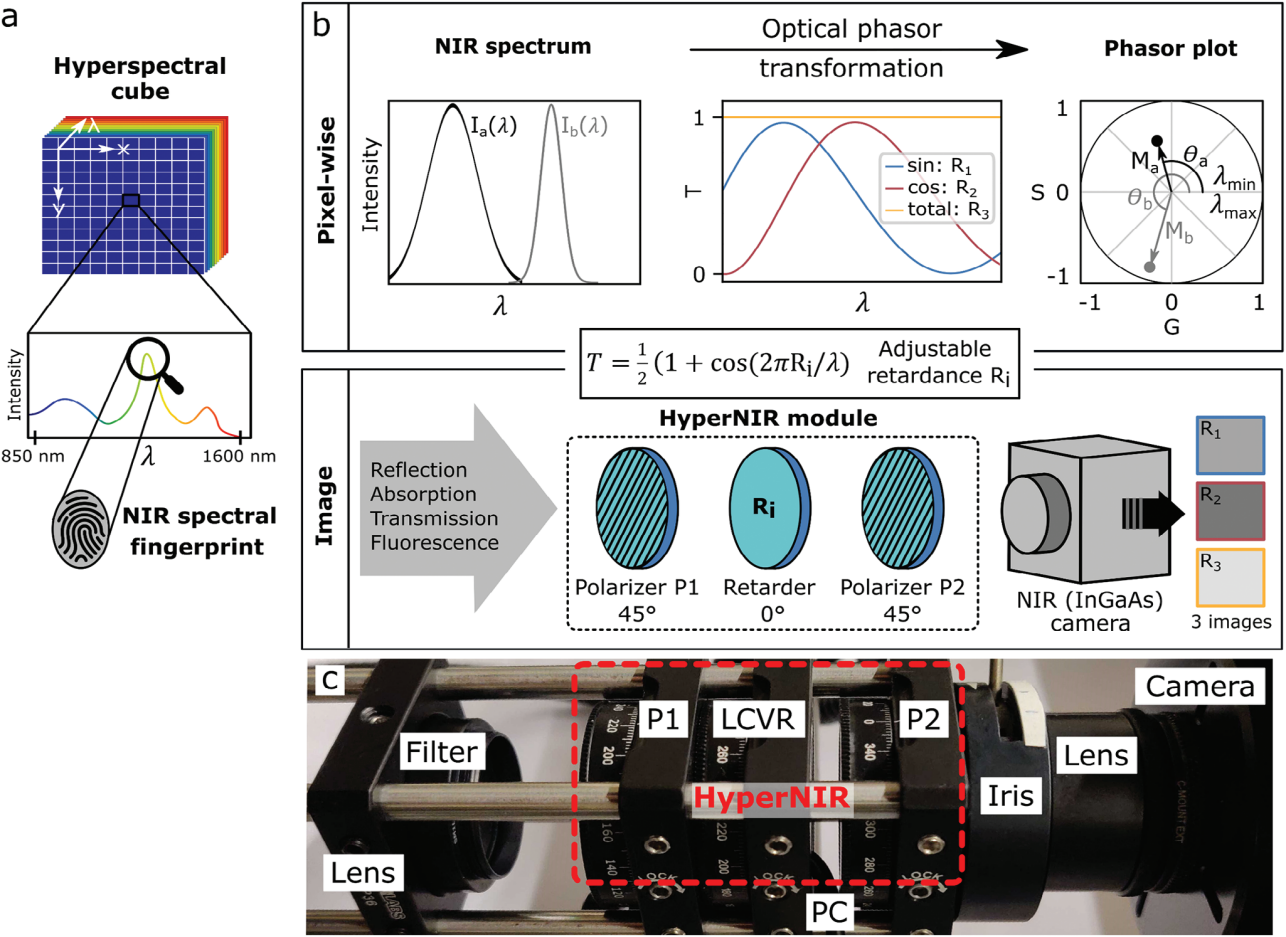
Implementation of phasor‐based tunable hyperspectral NIR imaging. a) A hyperspectral image corresponds to a 3D data cube (*x*,*y*,*λ*). In the near infrared (NIR), it contains either chemical information from combination and overtone modes that are useful for fingerprinting molecules/materials or signals from fluorophores. b) The spectral signal *I*_i_ (*λ*) is transformed into the phasor space by measuring its intensity with a sine‐, a cosine‐shaped, and a total transmission filter *T*(*λ*). Thereby, spectral information of a single pixel is represented as a point in 2D phasor space (*G* = cosine component of the spectrum, *S* = sine component of the spectrum, *M*_i_ = modulation, *θ*_i_ = phase). For a full hyperspectral image, the NIR signals are transmitted through a tunable cosine/sine filter, which is generated by the HyperNIR module. It consists of a modular retarder between two parallel linear polarizers, which approximates cosine/sine/total transmission filters by changing the retardance. The covered spectral range *λ*_min_ – *λ*_max_ is tunable because the retardance can be adjusted to squeeze or extend the sine/cosine. Three intensity images are taken by an InGaAs camera at different retardance (R_1_, R_2_, R_3_) to obtain the phasor position for each single pixel of the image. c) Picture of HyperNIR module connected to a NIR (InGaAs) camera (P1 = polarizer 1, P2 = polarizer 2, LCVR = liquid crystal variable retarder, PC = connection to computer).

Combining spectral and spatial information (hyperspectral imaging) enables novel applications such as environmental monitoring using drones.^[^ ^27^ ^]^ In contrast to conventional imaging, hyperspectral imaging assigns a spectrum, or at least spectral information, to each pixel, creating a 3D data cube (**Figure** **1**a). Hyperspectral imaging is performed by either scanning spatially (pushbroom scanning) or spectrally, which makes it inherently slow.^[^ ^28^ ^]^ For the NIR region, serial imaging using volume Bragg gratings has been demonstrated.^[^ ^29^ ^]^ An alternative are snapshot cameras that capture spatial and spectral information in a single shot. Hyperspectral cameras consisting of a Bayer‐like mosaic pattern^[^ ^30^ ^]^ or utilizing the light field technology^[^ ^31^, ^32^ ^]^ can achieve high frame rates but with a compromise between spectral and spatial resolution (Table S1, Supporting Information). The number of pixels in NIR (InGaAs) cameras is typically very low (around 0.1 MP), thus keeping all camera pixels for a high resolution is desired. Methods based on reconstruction, such as Fourier coded aperture transform spectral imaging (FCTS), can compensate for spectral and spatial resolution, but their optical setup is more sophisticated and requires extensive computational reconstruction.^[^ ^33^, ^34^, ^35^, ^36^, ^37^ ^]^

One alternative is to determine a spectral phasor for each pixel, which maps each pixel into a 2D (phasor) space, and the position contains the spectral information.^[^ ^38^ ^]^ The phasor approach is well known for interpreting fluorescence lifetime images. It provides a fit‐free analysis of time‐resolved data, which simplifies the analysis of complex samples.^[^ ^39^ ^]^ There is also a counterpart in the spectral domain, which requires images acquired at different wavelengths or the full spectrum of each pixel.^[^ ^38^ ^]^ This method finds wide application in Vis fluorescence imaging as well as in Raman hyperspectral imaging in the mid‐IR.^[^ ^40^, ^41^, ^42^ ^]^ Typically, the spectrum is measured using a scanning approach, and afterward, a phasor analysis is used to simplify the complexity of the data set. Instead of computing the phasor transformation from a measured spectrum, the same information can be obtained in a direct optical approach based on appropriate (hardware) filters.^[^ ^43^ ^]^ So far, commercially available absorption filters with a poor fit to sine/cosine^[^ ^43^, ^44^ ^]^ or custom‐made interference filters^[^ ^45^, ^46^, ^47^ ^]^ have been employed to implement direct phasor‐acquisition in the Vis range. As the filters are made for a fixed wavelength range, they cannot be adapted to different spectral regions and ranges. Overall, the potential of direct phasor‐acquisition has not been leveraged yet for hyperspectral imaging and the NIR range of the spectrum is especially challenging.

Here, we present a straightforward and tunable implementation of direct phasor acquisition (Figure 1b). Using this approach, we showcase NIR hyperspectral imaging of fluorophores as well as absorption signals from different plastics and monitor the water uptake of plants in a spatiotemporal fashion.

## 2 Results

### 2.1 Implementation of HyperNIR

To implement a tunable wavelength range, we used polarization optics to generate cosine‐ or sine‐shaped spectral transmissions. The optical module (HyperNIR) consists of two parallel linear polarizers and a variable retarder (Figure 1b). Both linear polarizers operate at an angle of 45° with respect to the fast axis of the variable retarder. NIR light from fluorescence, reflected or transmitted signals, reaches the first polarizer. In most cases, this light is randomly polarized but could be partially polarized due to polarization‐dependent reflection, absorption, or slow orientational dynamics of fluorophores. In either case, the first linear polarizer filters the 45° component. After the first polarizer, the retarder imposes a variable retardation, which creates an elliptically polarized beam depending on the wavelength. With the second linear polarizer, the 45° component of the elliptically polarized light is filtered out. The overall effect of the two polarizers sandwiched around the retarder is to create a variable and wavelength‐dependent transmission that can be easily tuned by varying the retardance *R*_i_. Based on the Jones calculus, the wavelength dependent transmission *T*(*R*, λ) can be calculated (for details see Section S2, Supporting Information):

(1) $$T\;\left(R,\lambda \right)=\frac{I}{I_{0}}\;=\frac{1}{2}\;\left[1+\cos\left(2\pi R_{i}/\lambda \right)\right]$$

which only depends on *R*_i_ (and the λ). The retardance is adjustable by changing the term *R*  =  *d*Δ*n*(λ). Here, *d* represents the thickness of the retarder and Δ*n*(λ) is the birefringence of the material. By modulating one of these parameters, the cosine transmission behavior can be spectrally shifted. Therefore, for a specific selected range, the cosine can be transformed into a sine‐like shape. In addition, to calculate the spectral phasor, a normalization is needed. This normalization is achieved by setting the retardance term *d*Δ*n*(λ) to 0, for which transmission does not depend on the wavelength, resulting in *T*  =  1 over the whole spectral range.

In theory, the sine‐/cosine‐shaped transmission filter can be set to cover any wavelength range by changing the retardance of the variable retarder. However, in practice this is restricted by the retardance range of the retarder and the transmission of optical components as well as the spectral camera sensitivity. The wavelength range can be shifted or compressed and stretched (Figure S1a–f, Supporting Information), which enables a spectral zoom‐in or zoom‐out. However, when using this approach, there is a slight error in the calculation of the phasor point. The transmission does not follow a perfect cosine wave. Instead, the variable retarder is generating a cos(1/λ) function. This results in a shift of the phasor points depending on the magnitude of the error with respect to the perfect sine/cosine function (Figure S1d–f, Supporting Information). By tuning the retardance, the error can be minimized for the desired spectral region (Figure S1g–i, Supporting Information). If the phasor would be calculated in terms of frequency instead of wavelength, a perfect cosine wave would be achieved. Another benefit is the low‐cost (starting at 2000 €) and availability of its standard components (Figure 1c) compared to pricey custom‐built sine/cosine interference filters (Table S2, Supporting Information). We chose a liquid crystal variable retarder (LCVR) as the main element because retardance can be easily adjusted by applying a certain AC voltage (Figure S2a,b, Supporting Information), but other optical retarders such as Soleil–Babinet compensators could be used as well. The LCVR can be electronically switched from sine to cosine to no spectral filtering, which eliminates the need for mechanically switching filters.

**Figure 2:**
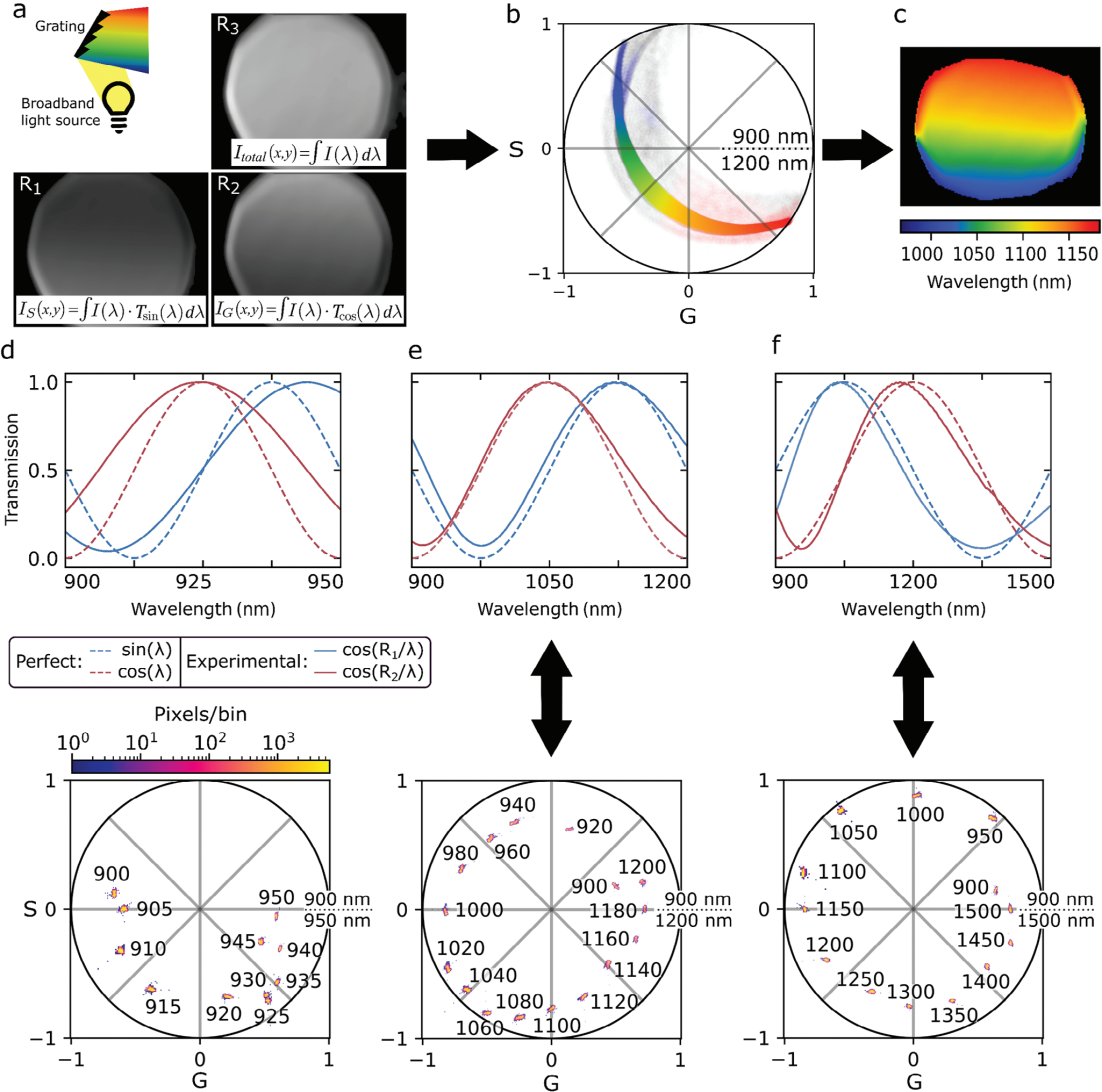
Phasor‐based hyperspectral imaging with polarization optics. Workflow of hyperspectral imaging. a) Model system based on diffraction of a (NIR) white light source from a grating (1200 L mm^−1^). Three images are collected at different retardances (R_1_, R_2_, R_3_), which results in a cosine image (*I*_G_(*x*,*y*)), a sine image (*I*_S_(*x*,*y*)) and a total intensity image (*I*_total_(*x*,*y*)). b) Every pixel is transformed into a point in the 2D phasor space. The position of the pixels in the phasor space contains spectral information and can be assigned to a colormap. c) Final HyperNIR image in which the colormap represents the angular position in the phasor plot according to their color in (b). Calibration measurements with a monochromatic light source (FWHM = 15 ± 2 nm) for different spectral ranges: d) 900–950 nm, e) 900–1200 nm, f) 900–1500 nm. Upper row: Best fit of the experimentally measured transmission curves to a perfect sine/cosine normalized to the maximum (dashed line). Lower row: Corresponding phasor plots. Input peak wavelengths of the monochromator are shown in nanometers.

The workflow for acquiring a hyperspectral image is always the same (**Figure** **2**a–c). For demonstration purposes, a broadband NIR light and a grating of a monochromator setup (with an opened slit) were used to create an image of diffracted light (Figure 2a). To get the spectral phasor, three images are taken with the HyperNIR module in front of a standard InGaAs NIR camera: one with the sine‐shaped filtering (*T*_sin_(*R*_1_,λ)), one with a cosine‐shaped filtering (*T*_cos_(*R*_2_,λ)), and one with full transmission (*T*_total_(*R*_3_,λ)) for normalization. In each pixel (*x*,*y*) the integral of the transformed spectrum is measured. This makes it possible to calculate a phasor point with the coordinate (*G*,*S*) for each individual pixel (details in Section S1, Supporting Information):^[^ ^43^ ^]^

(2) $$\begin{array}{ccc} & & S\;\left(x,y\right)=2\;\frac{\int_{\lambda_{\min}}^{\lambda_{\max}}I\left(\lambda ,x,y\right)\cdot T_{\sin}\left(R_{1},\lambda \right)\;d\lambda }{\int_{\lambda_{\min}}^{\lambda_{\max}}I\left(\lambda ,x,y\right)\;\cdot T_{\mathrm{total}}\left(R_{3},\lambda \right)\;d\lambda }-1\\ & & =2\;\frac{I_{S}\left(x,y\right)}{I_{\mathrm{total}}\left(x,y\right)}-1 \end{array}$$

(3) $$\begin{array}{ccc} & & G\;\left(x,y\right)=2\;\frac{\int_{\lambda_{\min}}^{\lambda_{\max}}I\left(\lambda ,x,y\right)\cdot T_{\cos}\left(R_{2},\lambda \right)\;d\lambda }{\int_{\lambda_{\min}}^{\lambda_{\max}}I\left(\lambda ,x,y\right)\;\cdot T_{\mathrm{total}}\left(R_{3},\lambda \right)\;d\lambda }-1\\ & & =2\;\frac{I_{G}\left(x,y\right)}{I_{\mathrm{total}}\left(x,y\right)}-1 \end{array}$$

Note that the 2 and 1 terms are necessary because of Equation (1) to normalize *G* and *S* and map the phasor onto a unity circle (values between −1 and 1). In summary, the phasor is represented as a scatter plot in which pixels with the same spectrum of light overlay (Figure 2b). Depending on the point's position, a color can be assigned. As a result, the plain intensity images are transformed into a pseudo‐color hyperspectral image (Figure 2c).

### 2.2 Tunable Phasor‐based Spectral Information

To characterize the image‐based phasor for different wavelengths, a monochromator with a xenon‐arc lamp was used. Its output was randomly polarized light, with a Gaussian spectrum and a full width at half maximum (FWHM) of 15 ± 2 nm. The tunable retardance allows to create different spectral ranges (Figure 2d–f), e.g., from 900 to 950 nm, 900 to 1200 nm, or 900 to 1500 nm. To obtain the best fit to a perfect sine and cosine wave in the specific wavelength regions, the spectral transmissions depending on the LCVR voltage were measured beforehand with a spectrophotometer (Figure S3d, Supporting Information). The transmission was normalized to the transmission performance of the optical components, which also included the 50% loss of intensity after the first polarizer, due to the random input polarization (Figure S3c, Supporting Information). For wavelength ranges narrower than 100 nm, the transmission fit became poorer (at a phasor range of 50 nm: *r* ^2^ = 0.82 for sine and *r* ^2^ = 0.96 for cosine) because of the restricted retardance range of the used LCVR (max *R* = 10.4 µm). In the related phasor plots below the transmission curves, it is possible to differentiate clusters. When the spectral range was decreased, the spectral resolution increased (for theoretical limitations see Section S3 and Figure S2, Supporting Information). The monochromatic 5 nm steps (Figure 2d) were equally well separated in the phasor plot as those of 20 nm (Figure 2e) or 50 nm (Figure 2f). The spectra with peaks at 1045, 1050, and 1055 nm were indistinguishable in the range 900 to 1200 nm (Figure S4, Supporting Information). By zooming into a phasor range of 100 and 50 nm, the phasors of the spectra were decomposed.

For the range 900 to 1200 nm, the same measurement was repeated with a custom‐built interference filter set, which had a nearly perfect match to the sine and cosine function in this range (Figure S5, Supporting Information). As expected, in this case the clusters were lying on the edge of the phasor circle and were uniformly distributed, which represents an even spectral resolution in every part of the phasor circle. The tunable polarization approach therefore has the tradeoff of a certain difference/error to the perfect phasor position. To get the true phasor position, it would be possible to correct it by creating a look‐up table for the *G* and *S* values or calibrating with NIR standards.

### 2.3 Hyperspectral Imaging of NIR Fluorescence Signals

**Figure 3:**
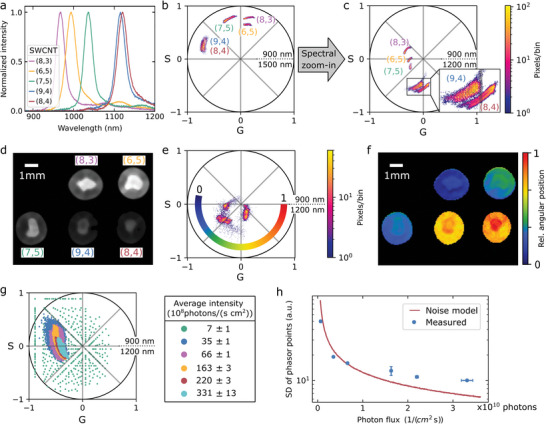
Hyperspectral imaging of NIR fluorescence signals. a) NIR fluorescence spectra of SWCNTs of different chiralities (*n*,*m*). b) Phasor plot of the SWCNTs from (a) in a homogenous solution in a broad wavelength range from 900 to 1600 nm. c) Spectral zoom‐in to the phasor range 900–1200 nm (by adjusting the retardance of HyperNIR) allows to differentiate (9,4)‐ and (8,4)‐SWCNTs. d) NIR fluorescence image of SWCNTs drop‐casted in small spots on a polymer membrane. e) HyperNIR transformation into the spectral phasor space. f) Based on the angular position in the phasor plot in (e), the pixels are color‐coded. g) Phasor plot for different levels of photon flux. h) Phasor uncertainty decreases with average light intensity (blue). The red line corresponds to a camera noise model (EMVA Standard 1288, see Experimental Section for details). Data = Mean ± SD, *n* = 3.

To demonstrate the (fluorescence) imaging capabilities, the HyperNIR module was placed in front of the camera port of a standard wide‐field microscope. As fluorophores, we chose semiconducting single‐walled carbon nanotubes (SWCNTs), which exhibit size‐dependent fluorescence between 870 and 2400 nm.^[^ ^48^ ^]^ They can be conceptualized as a rolled‐up single layer of graphene. Their structure is described by their roll‐up vector/chirality (*n*,*m*) and determines their absorption and emission spectrum, which makes them ideal NIR model systems and standards. We selected five of these SWCNTs: (8,3), (6,5), (7,5), (9,4), and (8,4), diluted in 1% sodium deoxycholate (DOC) in water (absorption spectra in Figure S6a, Supporting Information) with emission peaks in the range from 900 to 1200 nm (**Figure** **3**a). The (8,3)‐, (6,5)‐, and (7,5)‐SWCNTs showed distinct phasor clusters, which correspond to their emission spectra (Figure 3b). Due to the slightly broader emission of the (6,5)‐SWCNTs compared to the (8,3)‐SWCNTs, the pixels were closer to the center of the phasor plot. For the 900–1500 nm spectral phasor range, (9,4)‐ and (8,4)‐SWCNTs were not distinguishable. Thus, a spectral zoom‐in into the range 900–1200 nm was performed (Figure 3c) by adjusting the spectral range of HyperNIR. Consequently, the phasor clusters of (9,4)‐ and (8,4)‐SWCNTs were separated despite having nearly identical fluorescence spectra with center wavelengths only 5 nm apart. By reducing the phasor range from 600 to 240 nm the distance between the mean values in the phasor plot could be increased by 100% (Figure S7, Supporting Information). These results show the advantage of using a tunable filter instead of a fixed hardware filter.

The five SWCNT chiralities were also drop‐casted on a polymer membrane (Figure S6b, Supporting Information). The array was imaged with the same microscope using a lens (*f* = 150 mm, de‐magnification 0.75×) in place of the objective (Figure 3d). In contrast to the phasor plot of the solutions, the absolute position of the phasor was shifted for each chirality (Figure 3e). A reason for this could be the background signal of the membrane, which was subtracted by measuring the phasor of the membrane. We then created a color‐coded hyperspectral image based on the angular position of the pixels in the phasor (Figure 3f). Additionally, spectral differences in single spots were visible that are most likely caused by inhomogeneous drying of SWCNTs and aggregation, which is known to red‐shift/broaden SWCNT spectra.^[^ ^39^ ^]^ The SWCNT emission can be tuned by introducing guanine quantum defects (from 990 to 1040 nm), which results in a peak shift and broadening^[^ ^41^ ^]^ (Figure S8a, Supporting Information). Such changes can be monitored with HyperNIR, and the composition of samples with SWCNTs with and without defects could be determined based on the phasor position (Figure S8b and Table S3, Supporting Information).

To understand the limits of HyperNIR at low light intensities, a solution of (6,5)‐SWCNTs was imaged at six different concentrations (Figure 3g). The smaller the average intensity became, the greater was the spread of the phasor points. For an average intensity of about 10^9^ photons cm^−^^2^ s^−1^, the noise of the InGaAs camera resulted in a broad distribution in the phasor. Compared to the other five data points, there was no cluster observable. We then calculated the sum of the *S* and *G* standard deviations for the *G* and *S* values and plotted it against the average intensity in the image (Figure 3h). It is well fitted by a noise model for cameras based on the “EMVA Standard 1288”^[^ ^49^ ^]^ (see Experimental Section).

These results show that there is a photon limit of ≈10^9^ photons s^−1^ cm^−^^2^ to accurately determine the phasor of an image. This limit reflects the expected uncertainty for wavelength measurements of signals with low photon statistics and is close to photon limits for wide‐field fluorescence microscopy reported in literature.^[^ ^50^ ^]^ The fluorescence quantum yields of NIR fluorophores are typically low^[^ ^51^ ^]^ and therefore this is more relevant in low photon number fluorescence experiments than NIR reflection/absorption measurements with many photons.

### 2.4 Macroscopic HyperNIR Reflectance Imaging

Next, we tested HyperNIR reflectance imaging of macroscopic samples. The samples were illuminated by a tungsten‐halogen lamp at an approximate angle of 70° (Figure S9a, Supporting Information). The diffuse reflected light was then imaged through two demagnifying lenses (*M* = 0.15×). As a homogeneous reference, a diffuse reflectance standard with a near‐perfect Lambertian reflection was utilized. To cover the full spectral reflection of the NIR, the phasor range was chosen from 900 to 1600 nm. At first the reference was measured for each of the three retardance settings. We observed certain inhomogeneous intensity patterns (Figure S9b, Supporting Information) caused by an imperfect illumination and aberrations. By calculating a generic correction matrix for the *G* and *S* components, a dependence on the LCVR setting became visible (Figure S9c, Supporting Information). This inhomogeneity is well known in liquid crystal variable retarders,^[^ ^52^, ^53^ ^]^ but by applying the correction matrices, this effect is suppressed.

**Figure 4:**
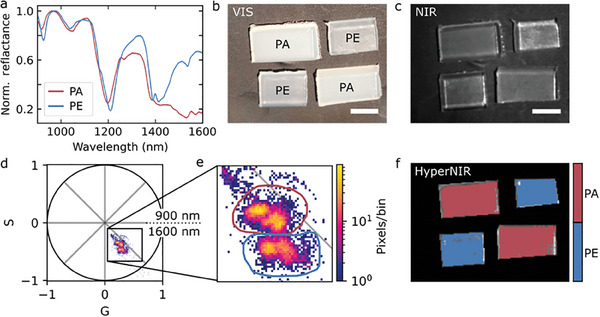
Hyperspectral NIR discrimination of plastics. a) NIR reflectance spectra of the plastic polymers polyethylene (PE) and polyamide (PA). b) Visible image of pieces of PA and PE. c) Corresponding NIR intensity image. d) Corresponding HyperNIR phasor plot for the range 900–1600 nm. e) Magnified plot of the phasor points in (d). f) HyperNIR image with adual‐color coding based on the pixel position in the phasor plot according to the blue and red circles in (e). Scale bar = 0.5 mm, exposure times (NIR & HyperNIR) = 15 ms.

The identification and differentiation of plastics is highly relevant in waste management and recycling applications.^[^ ^54^ ^]^ To demonstrate the capabilities of HyperNIR, we imaged two different plastics, namely polyethylene (PE) and polyamide (PA), next to each other. The reflectance spectra were very similar besides differences in the region from 1400 to 1600 nm (**Figure** **4**a). In the visible spectral range, the color of the pieces was nearly the same (Figure 4b), which was also found for the monochrome NIR image (Figure 4c). For a better classification, we calculated the phasor for each pixel (Figure 4d) and observed two separate clusters (Figure 4e). We then applied a polygon mask to color code the pixels in the image accordingly (Figure 4f). We also imaged with multi‐order sine and cosine filters (full wave for the range 900–1200 nm and for 1100–1600 nm, Figure S10a, Supporting Information). In this case the phasor is not unambiguous, but between 900 and 1200 nm the reflectance spectra showed no significant spectral differences. Therefore, this range did not contribute to a difference in the phasor as much as the 1100–1600 nm range, for which distinct peaks and an overall difference in the spectra was observed. By applying this filtering, we again found two clusters in the phasor space, which was used for binary color coding (Figure S10b–f, Supporting Information). This example shows that even without additional bandpass filters, it is possible to use different spectral ranges of HyperNIR. Overall, we show that HyperNIR enables fast discrimination of plastic polymers from 2D images, which is an advantage compared to using 1D hyperspectral cameras in combination with the necessity of moving the sample.^[^ ^55^ ^]^

**Figure 5:**
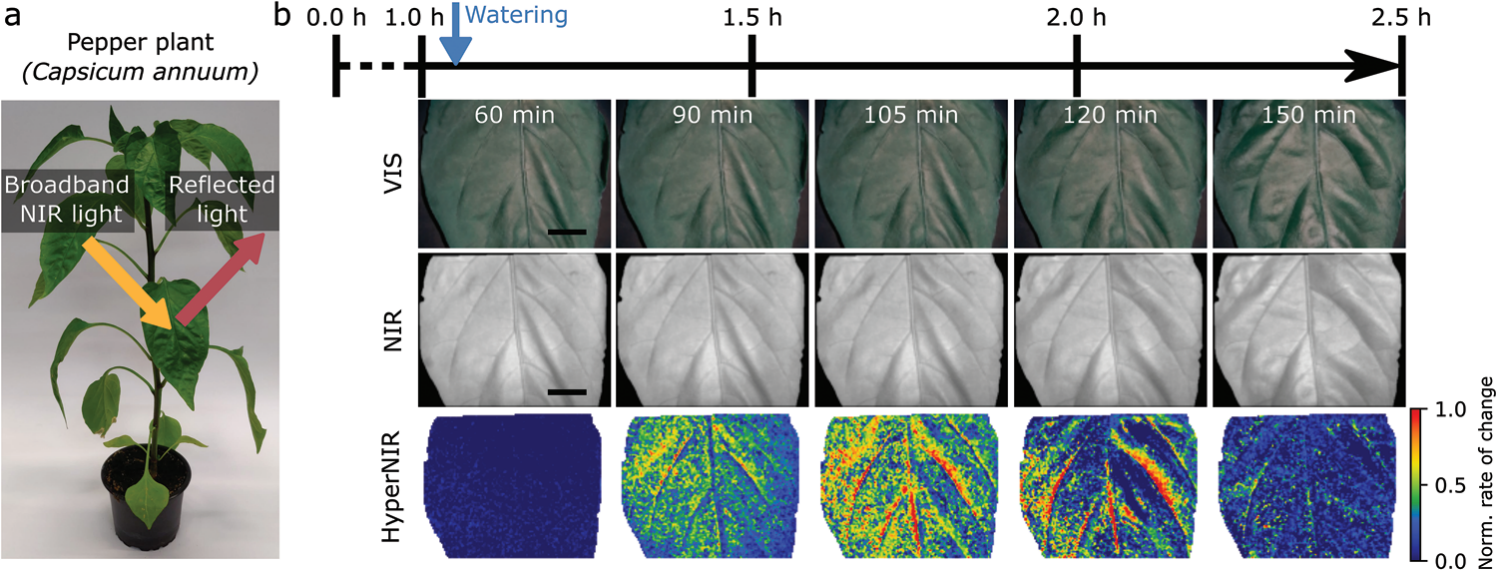
NIR hyperspectral imaging of leaf water uptake. a) Pepper plant (*Capsicum annuum*) and design of label‐free reflectance imaging. b) The RGB camera (VIS), NIR‐monochrome (NIR), and HyperNIR image from a leaf of a dry plant were captured over 2.5 h with a hyperspectral frame rate of 0.2 s^−1^ (images were taken from the Video S1, Supporting Information). After ≈61 min the plant was watered. In the HyperNIR images, the normalized rate of change is visualized, which represents the movement of the phasor relative to the phasor position at the beginning. It represents leaf water uptake based on the water combination modes in the NIR. Scale bar = 1 mm, exposure time (NIR & HyperNIR) = 7 ms.

Next, we wanted to explore the potential of HyperNIR for imaging of biological systems. Tools that monitor plant stress are necessary for precision agriculture.^[^ ^56^ ^]^ Water is essential for plants and has prominent features in the NIR. Therefore, we chose to monitor water uptake in plants. Plant leaves show high reflection in the range from 900 to 1350 nm, which corresponds to light scattering at the spongy cell structure of the leaves.^[^ ^57^ ^]^ At around 1450 nm, the water absorption peak dominates the spectrum (Figure S11, Supporting Information). By using this feature in a phasor, we wanted to measure the spatial and temporal distribution of the water uptake inside a leaf. As a model system, we used pepper plants (*Capsicum annuum*, **Figure** **5**a), which were not watered for 3 days. One leaf of the dry plant was placed in the field‐of‐view and imaged with a frame rate of 0.2 hyperspectral cubes per second. This frame rate is limited by the switching times of the LCVR between the three retardance settings (Figure S12, Supporting Information). We observed weak intensity fluctuation between two pictures (0.2 ± 0.4% over all camera pixels, see Figure S13, Supporting Information), most likely limited by the noise level of the InGaAs camera. The relative change of the phasor (Euclidean distance) in relation to the start of the measurement was calculated for each time point and color‐coded (Figure 5b and Video S1, Supporting Information). The plant was watered 61 min after the video started, but the first 15 min after watering showed nearly no change (only random fluctuations) in the HyperNIR image (Video S1 and and S15, Supporting Information). 25 min after watering, water transport in the capillaries became visible, and after around 30–60 min, also the surrounding tissue showed changes in the phasor, indicating an increase of the water concentration in the tissue (Figure 5b and Figure S15, Supporting Information). Such patterns were not observed in control experiments without water exposure (Figure S14, Supporting Information). After 2 h the rate of change decreased, resulting in a nearly homogenous image (for other leaves/plants see Figure S16, Supporting Information). These results align well with previously reported visualization of water transport and spatial water redistribution in leaves.^[^ ^58^, ^59^, ^60^, ^61^ ^]^ They show that HyperNIR enables in vivo imaging of plant health without the need of dyes, and this could be extended to biochemical compounds that are linked to other health states such as lipids. Moreover, one could use this approach to monitor processes related to photosynthesis and assess plant productivity, which is highly interesting for precision agriculture.

## 3 Conclusion/Discussion

HyperNIR is a powerful contactless technique for hyperspectral NIR fluorescence or label‐free (reflectance) imaging. It uses mapping of spectral information into a 2D space (phasor) by imaging through different filters similar to a hardware‐based Fourier transformation. We demonstrate a tunable and straightforward optical approach to obtain the spectral phasor using polarization optics. By adjusting the retardance of the LCVR, we shifted the phasor range and distinguished NIR fluorophores with 5 nm resolution within a spectral range of 300 nm. This opens the possibility to perform a spectral zoom‐in and zoom‐out, which could also be useful to avoid bandpass filters in situations when the multi‐order cosine and sine filters are not unambiguous. Furthermore, this approach covers the full spectral range from 900 to 1600 nm, allowing spatial NIR spectroscopy. This is demonstrated by plastic differentiation and monitoring of water content in leaves. In terms of sensitivity, the setup reached signal‐to‐noise ratios from 29.1 ± 0.1 dB (fluorescence, *t*_exp_ = 3 s) to 53.4 ± 0.9 dB (reflectance, *t*_exp_ = 7r ms). The maximum achievable hyperspectral frame rate with HyperNIR is one third of the frame rate for monochromic images because one requires three images for the phasor calculation. In videos we achieved so far frame rates of 0.2 hyperspectral cubes per second. The limiting factor for acquisition speed is the switching time of the LCVR. We used a commercially available one (Thorlabs), but faster models would directly translate into higher frame rates. Approaches such as splitting the LCVR in a few cells and driving every cell separately^[^ ^62^ ^]^ or using polymer‐stabilized or polymer‐dispersed liquid crystals can decrease the switching times.^[^ ^63^, ^64^, ^65^ ^]^

Recent advances in hyperspectral imaging leverage scanning techniques by motion‐guided reconstruction^[^ ^66^ ^]^ or fast acousto‐optic filtering,^[^ ^67^ ^]^ also overcome previous limitations in frame rates and spectral resolution. While our method has so far lower temporal resolution, it offers significant advantages in simplicity, cost‐effectiveness, and real‐time computational efficiency. The used optical elements are readily accessible and can be bought below 2000 € (Table S1, Supporting Information). The only change in an optical experiment is to place the HyperNIR module in front of the camera. Therefore, HyperNIR can be seen as a straightforward add‐on to equip normal cameras with very fast hyperspectral imaging capabilities. Novel methods^[^ ^68^, ^69^ ^]^ operating in the InGaAs range use on‐chip filter technologies to encode the spectral image and can maintain great spectral and spatial resolution. While these methods require post‐imaging reconstruction, our phasor‐based approach achieves continuous frame rates with minimal processing time. The computational assembly of the spectral image takes less than 5 ms on a standard laptop computer, which demonstrates the potential for fast live imaging.

The HyperNIR approach is also directly applicable to the Vis range of the spectrum if one uses the appropriate optical components. However, the NIR contains more relevant spectral information for label‐free fingerprinting. Additionally, the spectral Vis range is smaller, and typically Si‐based Vis cameras offer a much higher resolution (number of pixels). Therefore, the NIR is the spectral window in which HyperNIR can use and leverage its advantages. Another option is to use Vis‐SWIR cameras to further increase the spectral range and the information content of the images.

As we showed, HyperNIR can be used for fluorescence imaging as well as for label‐free imaging. Fluorescence imaging in this range is especially useful in the biomedical field, and spectral multiplexing is desired to increase the information content or observe wavelength shifts.^[^ ^26^ ^]^ NIR spectroscopy is often used as a fast method to identify materials, and our approach could make the imaging counterpart available for a much bigger community. Moreover, one can anticipate that using 2D chemometrics and machine learning approaches will allow tailored analysis of HyperNIR images and videos in the future.

Overall, we present a powerful method to obtain hyperspectral information by attaching a small‐footprint HyperNIR module to a camera. This could be especially relevant for experiments and applications in which a low mass and volume are desired, such as drones. In conclusion, we anticipate great potential for fast NIR hyperspectral imaging. It can pave the way for imaging of multiple NIR fluorophores or for label‐free imaging for environmental monitoring and precision agriculture.

## 4 Experimental Section

### HyperNIR Module and Acquisition

The HyperNIR module consists of two linear polarizers (LPNIR100, Thorlabs) and a liquid crystal variable retarder (LCC1115‐B, Thorlabs), which was operated by an LC‐controller (KLC101, Thorlabs). For all measurements, the frequency of the LCVR was kept at 5 kHz. For acquisition, the LCVR switching process was automated via the Python SDK. As an NIR camera, an InGaAs camera (Xeva 1.7 320 TE3 USB 100, Xenics) with an image format of 256 × 312 pixels was used. For the acquisition, the LCVR was controlled by the Python SDK (K‐Cube LC Controller Software, Thorlabs), and the camera was operated with “µManager” and the Python package *pycromanager*. The *G* and *S* values were calculated in Python with regard to Equations (2) and (3) with minor adjustments (Section S2, Supporting Information). The phasor plots were created using a 2D‐histogram (*matplotlib.pyplot.hist2d*) with bin sizes adjusted to the amount of analyzed pixels.

### Transmission Measurement and LCVR Voltage Settings

Transmission spectra were acquired with a spectrophotometer V‐780 (JASCO Deutschland GmbH). With a customized 3D‐printed adapter, the 30 mm cage system of HyperNIR was centered in the light path. The transmission was measured for the wavelength range 750–1600 nm. The measured curves were normalized by the transmission performance of the two polarizers and the LCVR (Figure S2c, Supporting Information). The applied voltage at the LCVR was changed from 0 to 4 V in 0.005 V steps, and subsequently the transmission spectrum was recorded. The stack of measured transmission curves was correlated with a perfect sine and cosine function for a defined spectral phasor range. The best fit was calculated by a Python script using the Pearson correlation. The curves with the highest r‐value were then used for the phasor acquisition. For the best fit, both negative and positive cosine and sine functions were evaluated. If a negated cosine or sine represents the best fit, this was considered in the phasor transformation (Section S2, Supporting Information).

### Characterization with Monochromatic Light Setup

The monochromatic source was powered by a xenon‐arc lamp (300 W) and split into spectral bands by a monochromator (MSH‐150, LOT‐Quantum Design GmbH, f/4.6, f = 150 mm) using a diffraction grating (MSG‐T‐1200‐500, 30 mm × 30 mm, 1200 L mm^−1^). The light was then passed through the polarization optics and was collected by a liquid light guide (Ø5 mm core, LLG5‐4Z, Thorlabs), which was coupled out by a collimator (SLSLLG1, Thorlabs) into an Olympus IX73 inverted microscope (Objective: Olympus MPlan N, ×5/NA 0.10). The tube lens of the microscope focused the light onto the InGaAs camera. The image of the diffracted light was post‐processed by subtracting a background signal and setting a threshold. To suppress pixel errors, the image was filtered with a median filter (*scipy.signal.medfilt2d*, filter window = (3,3)). Pixels with phasor points lying outside of the unity circle were ignored for the color‐coded image. Based on the phasor points angle, a colormap was applied. For the monochromatic signals, the same post‐processing was done.

### NIR Fluorophore Preparation

Single‐walled carbon nanotubes (SWCNT) were used as versatile NIR model fluorophores. As previously reported,^[^ ^70^, ^71^ ^]^ the separation of chiralities (9,4)‐, (7,5)‐, (8,4)‐, (8,3)‐, and (6,5)‐SWCNTs was achieved using an aqueous two‐phase extraction (ATPE) method. Initially, a raw (6,5) chirality enriched SWCNT mixture (CoMoCat, Sigma‐Aldrich) was suspended in a solution containing dextran (MW 70 000 Da, TCI) and polyethylene glycol (PEG, MW 6000 Da, Sigma‐Aldrich) to create two distinct aqueous phases. A fixed concentration of sodium deoxycholate (DOC, 0.05% m/v, Sigma‐Aldrich) and gradually increasing concentrations of sodium dodecyl sulfate (SDS, 0.5–1.5% m/v, Sigma‐Aldrich) were employed. Subsequently, a semiconducting‐metallic sorting step removed metallic SWCNTs from the chiral‐enriched fractions.^[^ ^70^ ^]^ In this step, sodium cholate (SC) was added, and the overall surfactant concentrations were adjusted to 0.9% SC, 1% SDS, and less than 0.02% DOC, followed by the addition of sodium hypochlorite (NaClO, 10–15% available chlorine, Honeywell) at 5 µL mL^−1^ of a solution prediluted to a 1/100th concentration in water as the oxidant. All sorted species were reconcentrated and adjusted to 1% DOC (m/v) using iterative concentration and dilution cycles in a pressurized ultrafiltration stirred cell (Millipore) equipped with a 300 kDa cutoff membrane. The microscopic measurements were done by pipetting 150 µL of each SWCNT solution in a black 96 microtiter well plate with a clear bottom (Fisher Scientific). The SWCNT array was prepared on a polyvinylidene fluoride (PVDF) membrane, which has a low background fluorescence in the visible and NIR (Immobilon‐FL PVDF‐Membran, Milipore). For every SWCNT chirality, 1.5 µL were drop‐casted on the membrane and dried on a hot plate operated at 60 °C.

For the concentration‐dependent phasor, (6,5)‐enriched SWCNTs without purification were used. Compared to the chirality‐pure SWCNTs, these SWCNTs were solubilized by biofunctionalization with the ssDNA sequence (GT)_15_. In short, (GT)_15_ ssDNA (200 µL, 100 µm in PBS, Sigma Aldrich) was mixed with SWCNTs (100 µL, 2 mg mL^−1^ in PBS) and tip‐sonicated (Fisher Scientific Model 120 Sonic Dismembrator, 35% amplitude, 20 min pulsed: 9 s on, 1 s off). The resulting suspension was centrifuged (2×, 21 000*g*, 30 min), and the supernatant was transferred into a different reaction vessel. The SWCNTs were prepared in phosphate buffered saline (1×PBS) in the concentrations of 0.1, 0.5, 1, 3, 5, and 10 nm.^[^ ^72^ ^]^ For the measurement, triplicates of 200 µL of each concentration were pipetted into a 96 microtiter well plate. Additionally, for background subtraction, triplicates of 200 µL 1×PBS were used.

The preparation of SWCNTs with guanine quantum defects (*G* ^d^) followed a previously published protocol.^[^ ^73^ ^]^ For the covalent functionalization of guanine‐containing ssDNA with the SWCNTs, the (GT)_15_‐functionalized SWCNTs (189 µL, 10.6 nm in PBS) were mixed with Rose Bengal (10.1 µL, 330 µm in H_2_O, Sigma‐Aldrich) in a 96‐microwell plate and irradiated (Analytical Sales Lumidox 96‐Well Green LED Array + LED Controller, 25 mA, ≈527 nm). After 10 min, air was bubbled through the mixtures, and the respective wells were irradiated for an additional 5 min. Free DNA and Rose Bengal were removed via dialysis (Spectrum Laboratories SpectraPor membranes, molecular weight cut‐off: 300 kDa) for five days with buffer changes twice a day. The suspensions were briefly tip‐sonicated for redispersion (35% amplitude, 20 s), centrifuged (21 000*g*, 20 min), and the supernatant was used for further studies. The *G* ^d^‐SWCNTs were mixed with chirality‐pure (6,5)‐SWCNTs, which both were prepared in the concentration of 1 nm. Mixtures of (6,5)‐SWCNTs without and with defects were prepared in ratios of 50:50, 75:25, 90:10, and 95:5 in a total volume of 200 µL. To gain the phasor of the pure samples, 200 µL of each were used as a reference.

### NIR Fluorophore Spectroscopy and Imaging

Emission spectra of SWCNTs were acquired with a custom‐build microscope setup, which was described in previous studies,^[^ ^73^ ^]^ equipped with a 561 nm laser (gem‐561, Novanta Photonics) and an InGaAs spectrograph (Shamrock 193i + Andor iDus InGaAs 491 detector, Andor Technology). Background signals were corrected in the spectrometer software using a spectrum of the buffer.

All SWCNT measurements were performed at the inverted microscope (Nikon Eclipse Ti2). It consisted of an excitation light (CoolLED pE300 Lite, 100% power), which was transmitted through a bandpass (for (6,5)‐SWCNTs: 560 ± 40 nm (F47‐561, AHF Analysentechnik) or for measurement with more SWCNT chiralities: 495 ± 130 nm (FSR‐BG40, Newport)) and an 804 nm longpass dichroic mirror (AHF Analysentechnik F38‐801) before being focused by an objective (CFI Plan Fluor DL 10XF, Nikon). Emission light passed through an 840 nm longpass filter (F47‐841, AHF Analysentechnik). The HyperNIR setup and Xeva camera were positioned at the output of the left camera port.

For all solution measurements: the focus was adjusted to approximately the middle of the sample in order to achieve maximal signal and a homogenous distribution. The exposure times for the images were set to 3 s. A background signal of the excitation source (LED) and dark noise was subtracted from the fluorescence image for each sine, cosine, and total image. Due to strong circular vignetting at the edges, a circular mask was applied. The images were filtered with a median filter (filter window = (3,3)). For the concentration‐dependent phasor analysis, the spreading of the phasor clusters was evaluated by calculating the sum of the standard deviation of the *G* and *S* components over all phasor points for each concentration. For spectral unmixing of SWCNTs with and without guanine quantum defects, the fractions were calculated according to Section S4 (Supporting Information).

For imaging the SWCNT array, a 150 mm lens (AC254‐150‐AB‐ML, Thorlabs) instead of a microscope objective was used. The remaining optical setup was the same as for the SWCNT solution imaging. As a background signal, a membrane without any SWCNTs were imaged. Due to the inhomogeneous drying of the samples, a stronger median filter (filter window = (9,9)) was applied.

### Noise Model

The used noise model is based on the “EMVA Standard 1288: Standard for Characterization of Image Sensors and Cameras.”^[^ ^49^ ^]^ The overall variance $\sigma_{y}^{2}\;$ of the detected signal at the camera is the linear sum of all noise sources:

(4) $$\sigma_{y}^{2}=K^{2}\;\sigma_{d}^{2}+\sigma_{q}^{2}+K\left(\mu_{y}-\mu_{y,\mathrm{dark}}\right)$$

where *K* is the system gain of the camera, $\sigma_{d}^{2}$ represents the read‐out noise, and $\sigma_{q}^{2}$ stands for the quantization noise. The noise is mainly influenced by the amount of signal µ_y_ reaching the detector, which is subtracted by the dark signal µ_y,dark_. For the used InGaAs camera, the parameters can be found in the data sheet: $\sigma_{d}^{2}=1000\;e^{-}$, $\sigma_{q}^{2}=\frac{1}{12}\;DN^{2}$ (12‐bit), *K*  =  1.1  ×  10^−3^DN/*e* ^−^. The dark signal µ_y,dark_ was neglected in the theoretical calculation since the measured intensities were corrected by the background and the dark signal. For the noise calculation, images with 256×312 pixels with a normal Gaussian random distribution (Python function *numpy.random.normal*) were generated. This was done for a signal µ_y_ in the range from 10 to 600 DN. For the sine and cosine image, a fraction of this signal intensity was used, and the fraction was calculated by integrating the transmitted sine and cosine part of the (6,5)‐SWCNT spectra over all wavelengths and normalized it to the total intensity. For a realistic intensity profile, a circular Gaussian mask was applied. The photon flux on the camera was calculated by

(5) $$\Phi =\frac{\mu_{y}}{K\;\mathrm{QE}\;A\;t_{\exp}}\;\left[\frac{\#\;\mathrm{photons}}{cm^{2}\;s}\right]$$

where QE  =  0.56 (at 990 nm) is the quantum efficiency, *A*  =  9  ×  10^−6^ cm^2^ the pixel area of the camera, and *t*_exp_ =  3 s the exposure time of the camera.

### Macroscopic Reflectance Imaging

For HyperNIR imaging of macroscopic samples, a custom‐built setup was used. The camera was mounted vertically on an aluminum strut profile. The polarization optics were placed in between a magnifying two‐lens system. On the object‐side a 200 mm plano‐convex lens (LA1708, Thorlabs) was used, whereas the image was generated by a 30 mm achromatic lens (AC254‐030‐AB, Thorlabs). This resulted in a magnification of 0.15× for the overall system. Based on this, a field of view of 51 mm × 64 mm was obtained. Before the imaging lens, an iris diaphragm (SM1D25, Thorlabs) was a placed to adjust the incident light and improve the overall image quality. To filter out the visible spectral range, an 840 nm longpass filter (AHF Analysentechnik F47‐841) was positioned between the object lens and the first polarizer. As a NIR illumination source, a quartz tungsten‐halogen lamp (QTH10/M, Thorlabs) illuminated the sample at an approximate angle of 70°. For imaging, short exposure times between 5 and 50 ms were used. A correction matrix for *G* and *S* (Figure S9b and Section S5, Supporting Information) was obtained by measuring a white diffuse reflectance standard (Spectralon Ø 31.75 mm, Labsphere). Therefore, the reflectance standard was moved to all angles of the field‐of‐view and the images were then mapped together. To suppress reflections from the background, a matte black aluminum foil (BKF12, Thorlabs) was used as a base for the samples.

The plastic pieces were purchased from PlasticsEurope Deutschland e.V. as a sample collection. The samples were pure polyamide and pure polyethylene pieces with a thickness of 2.5 mm. In the post‐imaging analysis, the four plastic pieces were cut out and the rest of the background was set to 0. The *G* and *S* values were corrected with a correction matrix obtained by a white reflectance standard (Figure S9b and Section S5, Supporting Information). Polygon masks were used to define clusters in the phasor plot and create binary colormaps.

For the leaf water uptake experiments, a pepper plant (*Capsicum annuum*) was used. It was bought as a seedling in a pot at a local gardening store. To measure the water uptake, the plant was not watered for three to five days. One leaf was placed in the camera view and imaged with a hyperspectral frame rate of 0.2 s^−1^ (acquisition cycle: set 1.94 V → pause 1.5 s → set 2.19 V → pause 0.5 s → set 25 V → pause 3 s → set 1.94 V, see Figure S11, Supporting Information). After imaging the dry leaf for 1 h, the plant was watered with a volume of ≈40 mL. After that, the video continued for an additional 1.5 h. A polygon mask was used to extract the parts of the image that contained a leaf, while the rest of the image was set to 0. An average phasor point for each pixel as a reference for a dry leaf was calculated over the hyperspectral frames of the first 5 min. These phasor points were used as reference positions for the upcoming frames. For each pixel, the movement of the phasor (Euclidean distance) in contrast to the reference position was calculated. Based on this, the rate of change was calculated by forming the linear regression (least squares) over a range of 10 min. Afterward, all values were normalized to the rate of change which was that at 99% of the cumulative histogram (calculated over all frames and pixels). All *G* and *S* values were again corrected by the correction matrices. The Vis images were acquired by a webcam (Logitech) with 1920 × 1080 pixels.

## Conflict of Interest

S.K. and J.S. are listed as inventors on a pending patent application describing the HyperNIR module presented in this study.

## Supporting information



Supporting Information

Supplemental Movie 1

## Data Availability

The data that support the findings of this study are available from the corresponding author upon reasonable request.

## References

[1] N. T. Shaked , S. A. Boppart , L. V. Wang , J. Popp , Nat. Photonics 2023, 12, 1031. 10.1038/s41566-023-01299-6 PMC10956740 38523771

[2] W. Choi , C. Fang‐Yen , K. Badizadegan , S. Oh , N. Lue , R. R. Dasari , M. S. Feld , Nat. Methods 2007, 9, 717. 10.1038/nmeth1078 17694065

[3] Y. Park , C. Depeursinge , G. Popescu , Nat. Photonics 2018, 10, 578.

[4] R. Oldenbourg , Methods Cell Biol. 1998, 61, 175. 10.1016/s0091-679x(08)61981-0 9891315

[5] M. R. Kole , R. K. Reddy , M. V. Schulmerich , M. K. Gelber , R. Bhargava , Anal. Chem. 2012, 23, 10366. 10.1021/ac302513f PMC3514576 23113653

[6] A. J. Walsh , K. P. Mueller , K. Tweed , I. Jones , C. M. Walsh , N. J. Piscopo , N. M. Niemi , D. J. Pagliarini , K. Saha , M. C. Skala , Nat. Biomed. Eng. 2020, 1, 77. 10.1038/s41551-020-0592-z PMC7854821 32719514

[7] L. V. Wang , S. Hu , Science 2012, 6075, 1458. 10.1126/science.1216210 PMC3322413 22442475

[8] T. J. Allen , A. Hall , A. P. Dhillon , J. S. Owen , P. C. Beard , J. Biomed. Opt. 2012, 6, 061209. 10.1117/1.JBO.17.6.061209 22734739

[9] H. Ewers , V. Jacobsen , E. Klotzsch , A. E. Smith , A. Helenius , V. Sandoghdar , Nano Lett. 2007, 8, 2263. 10.1021/nl070766y 17637017

[10] P. Kukura , H. Ewers , C. Müller , A. Renn , A. Helenius , V. Sandoghdar , Nat. Methods 2009, 12, 923. 10.1038/nmeth.1395 19881510

[11] G. Young , P. Kukura , Annu. Rev. Phys. Chem. 2019, 70, 301. 30978297 10.1146/annurev-physchem-050317-021247

[12] H. Kobayashi , C. Lei , Y. Wu , A. Mao , Y. Jiang , B. Guo , Y. Ozeki , K. Goda , Sci. Rep. 2017, 1, 12454. 10.1038/s41598-017-12378-4 PMC5622112 28963483

[13] L. Tong , J.‐X. Cheng , Mater. Today 2011, 6, 264.

[14] Y. Ozaki , C. Huck , S. Tsuchikawa , S. Balling Engelsen , Near‐Infrared Spectroscopy, Springer Nature, Singapore 2021.

[15] M. Jamrógiewicz , J. Pharm. Biomed. Anal. 2012, 66, 1. 22469433 10.1016/j.jpba.2012.03.009

[16] J. A. Prananto , B. Minasny , T. Weaver , Adv. Agron. 2020, 164, 1.

[17] S. A. D. M. Zahir , A. F. Omar , M. F. Jamlos , M. A. M. Azmi , J. Muncan , Sens. Actuators, A 2022, 338, 113468.

[18] B. G. Osborne , Near‐Infrared Spectroscopy in Food Analysis, vol. 1, Wiley, Hoboken, NJ, USA 2006.

[19] A. Sakudo , Clin. Chim. Acta 2016, 455, 181. 26877058 10.1016/j.cca.2016.02.009

[20] A. Spreinat , G. Selvaggio , L. Erpenbeck , S. Kruss , J. Biophotonics 2020, 1, 201960080. 10.1002/jbio.201960080 PMC7065629 31602799

[21] A. M. Smith , M. C. Mancini , S. Nie , Nat. Nanotechnol. 2009, 11, 710. 10.1038/nnano.2009.326 PMC2862008 19898521

[22] G. Hong , A. L. Antaris , H. Dai , Nat. Biomed. Eng. 2017, 1, 0010.

[23] R. Nißler , A. T. Müller , F. Dohrman , L. Kurth , H. Li , E. G. Cosio , B. S. Flavel , J. P. Giraldo , A. Mithöfer , S. Kruss , Angew. Chem., Int. Ed. Engl. 2022, 2, 202108373. 10.1002/anie.202108373 PMC9298901 34608727

[24] R. Nißler , O. Bader , M. Dohmen , S. G. Walter , C. Noll , G. Selvaggio , U. Groß , S. Kruss , Nat. Commun. 2020, 1, 5995. 10.1038/s41467-020-19718-5 PMC7689463 33239609

[25] S. Elizarova , A. A. Chouaib , A. Shaib , B. Hill , F. Mann , N. Brose , S. Kruss , J. A. Daniel , Proc. Natl. Acad. Sci. U. S. A. 2022, 22, e2202842119. 10.1073/pnas.2202842119 PMC9295782 35613050

[26] M. Kim , C. Chen , P. Wang , J. J. Mulvey , Y. Yang , C. Wun , M. Antman‐Passig , H.‐B. Luo , S. Cho , K. Long‐Roche , L. V. Ramanathan , A. Jagota , M. Zheng , Y. Wang , D. A. Heller , Nat. Biomed. Eng. 2022, 3, 267. 10.1038/s41551-022-00860-y PMC9108893 35301449

[27] T. Adão , J. Hruška , L. Pádua , J. Bessa , E. Peres , R. Morais , J. J. Sousa , Remote Sens. 2017, 11, 1110.

[28] B. Boldrini , W. Kessler , K. Rebner , R. W. Kessler , J. Near Infrared Spectrosc. 2012, 5, 483.

[29] D. Roxbury , P. V. Jena , R. M. Williams , B. Enyedi , P. Niethammer , S. Marcet , M. Verhaegen , S. Blais‐Ouellette , D. A. Heller , Sci. Rep. 2015, 1, 14167. 10.1038/srep14167 PMC4585673 26387482

[30] B. Geelen , N. Tack , A. Lambrechts , Proc. SPIE 2014, 8974, 89740L.

[31] Z. Xiong , L. Wang , H. Li , D. Liu , F. Wu , Proceedings of the IEEE Conference on Computer Vision and Pattern Recognition (CVPR), IEEE, New York City, USA, 2017, p. 3270.

[32] Y. Yoon , H.‐G. Jeon , D. Yoo , J.‐Y. Lee , I. S. Kweon , IEEE Signal Process. Lett. 2017, 24, 848.

[33] H. Xie , J. Lu , J. Han , Y. Zhang , F. Xiong , Z. Zhao , Opt. Lasers Eng. 2023, 163, 107443.

[34] M. Yako , Y. Yamaoka , T. Kiyohara , C. Hosokawa , A. Noda , K. Tack , N. Spooren , T. Hirasawa , A. Ishikawa , Nat. Photonics 2023, 3, 218.

[35] Y. Xu , L. Lu , V. Saragadam , K. F. Kelly , Nat. Commun. 2024, 1, 1456. 10.1038/s41467-024-45856-1 PMC10874389 38368402

[36] K. Monakhova , K. Yanny , N. Aggarwal , L. Waller , Optica 2020, 10, 1298.

[37] D. Mengu , A. Tabassum , M. Jarrahi , A. Ozcan , Light: Sci. Appl. 2023, 1, 86. 10.1038/s41377-023-01135-0 PMC10079962 37024463

[38] F. Fereidouni , A. N. Bader , H. C. Gerritsen , Opt. Express 2012, 12, 12729. 10.1364/OE.20.012729 22714302

[39] M. Bachilo , M. S. Strano , C. Kittrell , R. H. Hauge , R. E. Smalley , R. B. Weisman , Science 2002, 5602, 2361. 10.1126/science.1078727 12459549

[40] L. Scipioni , A. Rossetta , G. Tedeschi , E. Gratton , Nat. Methods 2021, 5, 542. 10.1038/s41592-021-01108-4 PMC10161785 33859440

[41] J. T. Metternich , J. A. C. Wartmann , L. Sistemich , R. Niβler , S. Herbertz , S. Kruss , J. Am. Chem. Soc. 2023, 27, 14776. 10.1021/jacs.3c03336 37367958

[42] W. J. Tipping , L. T. Wilson , C. An , A. A. Leventi , A. W. Wark , C. Wetherill , N. C. O. Tomkinson , K. Faulds , D. Graham , Chem. Sci. 2022, 12, 3468. 10.1039/d1sc06976d PMC8943890 35432863

[43] A. Dvornikov , E. Gratton , Biomed. Opt. Express 2018, 8, 3503. 10.1364/BOE.9.003503 PMC6191637 30338135

[44] J. E. Batey , M. Yang , H. Giang , B. Dong , Anal. Chem. 2023, 13, 5479. 10.1021/acs.analchem.2c05336 36883846

[45] P. N. Hedde , R. Cinco , L. Malacrida , A. Kamaid , E. Gratton , Commun. Biol. 2021, 1, 721. 10.1038/s42003-021-02266-z PMC8195998 34117344

[46] B. Torrado , A. Dvornikov , E. Gratton , Biomed. Opt. Express 2021, 7, 3760. 10.1364/BOE.422236 PMC8367243 34457378

[47] Z. Yao , C. K. Brennan , L. Scipioni , H. Chen , K. K. Ng , G. Tedeschi , K. Parag‐Sharma , A. L. Amelio , E. Gratton , M. A. Digman , J. A. Prescher , Nat. Methods 2022, 7, 893. 10.1038/s41592-022-01529-9 PMC12743604 35739310

[48] S. M. Bachilo , M. S. Strano , C. Kittrell , R. H. Hauge , R. E. Smalley , R. B. Weisman , Science 2002, 5602, 2361. 10.1126/science.1078727 12459549

[49] European Machine Vision Association, EMVA , Standard 1288: Standard for Characterization of Image Sensors and Cameras Release 3.1, European Machine Vision Association, Barcelona, Spain 2016.

[50] Y. Li , F. Huang , Nat. Commun. 2024, 1, 3760. 10.1038/s41467-024-48155-x PMC11069581 38704387

[51] A. Spreinat , M. M. Dohmen , J. Lüttgens , N. Herrmann , L. F. Klepzig , R. Nißler , S. Weber , F. A. Mann , J. Lauth , S. Kruss , J. Phys. Chem. C 2021, 33, 18341.

[52] J. Vargas , N. Uribe‐Patarroyo , J. Antonio Quiroga , A. Alvarez‐Herrero , T. Belenguer , Appl. Opt. 2010, 49, 568. 20119002 10.1364/AO.49.000568

[53] C. N. Ramírez , I. Montes‐González , N. C. Bruce , J. M. López‐Téllez , O. G. Rodríguez‐Herrera , M. Rosete‐Aguilar , Appl. Opt. 2021, 60, 2998. 33983193 10.1364/AO.418547

[54] J.‐P. Lange , ACS Sustainable Chem. Eng. 2021, 47, 15722.

[55] Y. Zheng , J. Bai , J. Xu , X. Li , Y. Zhang , Waste Manage. 2018, 72, 87. 10.1016/j.wasman.2017.10.015 29129466

[56] J. P. Giraldo , H. Wu , G. M. Newkirk , S. Kruss , Nat. Nanotechnol. 2019, 6, 541. 10.1038/s41565-019-0470-6 31168083

[57] D. M. Gates , H. J. Keegan , J. C. Schleter , V. R. Weidner , Appl. Opt., AO 1965, 1, 11.

[58] W. B. Herppich , C. E. Martin , C. Tötzke , I. Manke , N. Kardjilov , Plant, Cell & Environment 2019, 5, 1645. 10.1111/pce.13496 30506732

[59] T. Defraeye , D. Derome , W. Aregawi , D. Cantré , S. Hartmann , E. Lehmann , J. Carmeliet , F. Voisard , P. Verboven , B. Nicolai , Planta 2014, 2, 423. 10.1007/s00425-014-2093-3 24923675

[60] W. Liu , Y. Luo , L. Wang , T. Luo , Y. Peng , L. Wu , J. Plant Physiol. 2016, 204, 74. 27526337 10.1016/j.jplph.2016.06.022

[61] Z. Song , S. Yan , Z. Zang , Y. Fu , D. Wei , H.‐L. Cui , P. Lai , IEEE Trans. Terahertz Sci. Technol. 2018, 5, 520.

[62] H. Cheng , A. Bhowmik , P. J. Bos , Opt. Eng. 2013, 10, 107105.

[63] S. G. Kim , S. M. Kim , Y. S. Kim , H. K. Lee , S. H. Lee , G.‐D. Lee , J.‐J. Lyu , K. H. Kim , Appl. Phys. Lett. 2007, 26, 261910.

[64] D.‐K. Yang , L.‐C. Chien , J. W. Doane , Appl. Phys. Lett. 1992, 25, 3102.

[65] B.‐G. Wu , J. H. Erdmann , J. W. Doane , Liq. Cryst. 1989, 5, 1453.

[66] D. Li , J. Wu , J. Zhao , H. Xu , L. Bian , Nat. Commun. 2024, 1, 9459. 10.1038/s41467-024-53747-8 PMC11530457 39487117

[67] J. Fang , K. Huang , R. Qin , Y. Liang , E. Wu , M. Yan , H. Zeng , Nat. Commun. 2024, 1, 1811. 10.1038/s41467-024-46274-z PMC10902379 38418468

[68] L. Bian , Z. Wang , Y. Zhang , L. Li , Y. Zhang , C. Yang , W. Fang , J. Zhao , C. Zhu , Q. Meng , X. Peng , J. Zhang , Nature 2024, 8037, 73. 10.1038/s41586-024-08109-1 PMC11541218 39506154

[69] H. Meng , Y. Gao , X. Wang , X. Li , L. Wang , X. Zhao , B. Sun , Light: Sci. Appl. 2024, 1, 121. 10.1038/s41377-024-01476-4 PMC11130170 38802359

[70] J. A. Fagan , Nanoscale Adv. 2019, 9, 3307. 10.1039/c9na00280d PMC9417344 36133572

[71] H. Li , C. M. Sims , R. Kang , F. Biedermann , J. A. Fagan , B. S. Flavel , Carbon 2023, 204, 475.

[72] R. Nißler , F. A. Mann , P. Chaturvedi , J. Horlebein , D. Meyer , L. Vuković , S. Kruss , J. Phys. Chem. C 2019, 8, 4837.

[73] J. T. Metternich , J. A. C. Wartmann , L. Sistemich , R. Nißler , S. Herbertz , S. Kruss , J. Am. Chem. Soc. 2023, 27, 14776. 10.1021/jacs.3c03336 37367958

