# Supplementary material for: High‐Speed Hyperspectral Imaging for Near Infrared Fluorescence and Environmental Monitoring
Source: Adv Sci (Weinh). 2025 Mar 4;12(16):2415238. doi: 10.1002/advs.202415238 (PMC12021105; doi:10.1002/advs.202415238)
Supplement: Supplementary file 1 — Supporting Information [file ADVS-12-2415238-s001.pdf]

ADVANCED SCIENCE
Open Access

Supporting Information

for *Adv. Sci.*, DOI 10.1002/advs.202415238

High-Speed Hyperspectral Imaging for Near Infrared Fluorescence and Environmental Monitoring

*Jan Stegemann, Franziska Gröniger, Krisztian Neutsch, Han Li, Benjamin Scott Flavel, Justus Tom Metternich, Luise Erpenbeck, Poul Bering Petersen, Per Niklas Hedde and Sebastian Kruss**

Supporting Information

High-Speed Hyperspectral Imaging for Near Infrared Fluorescence and Environmental Monitoring

*Jan Stegemann, Franziska Gröniger, Krisztian Neutsch, Han Li, Benjamin Flavel, Justus Tom Metternich, Luise Erpenbeck, Poul Petersen, Per Niklas Hedde, Sebastian Kruss**

Table of Contents

Extended Methods **1**

1. Theory of the spectral phasor calculation 1
2. Theoretical background of the polarization filtering to create a cosine transmission 2
3. Phasor discrimination and spectral resolution 4
4. Spectral unmixing of NIR fluorophores 5
5. Correction for inhomogeneity in macroscopic images 6

Supplementary Videos **7**

Supplementary Figures and Tables **7**

References **19**

Extended Methods

1. Theory of the spectral phasor calculation

The phasor itself is the first harmonic of the Fourier transform of a spectrum $I(\lambda)$:[1]

$$\hat{F} = \int_{-\infty}^{\infty} I(\lambda) \cdot e^{-i\frac{2\pi}{L}\lambda}\, d\lambda \tag{1}$$

where $L = (\lambda_{max} - \lambda_{min})$ defines the analyzed spectral range. As a representation of the phasor, the real and imaginary parts of the Fourier transform G and S are used as coordinates to map the spectrum onto a 2D space:[1]

$$G = \frac{\int_{\lambda_{min}}^{\lambda_{max}} I(\lambda) \cdot cos\left(\frac{2\pi(\lambda-\lambda_{min})}{(\lambda_{max}-\lambda_{min})}\right) d\lambda}{\int_{\lambda_{min}}^{\lambda_{max}} I(\lambda)\, d\lambda} \tag{2}$$

$$S = \frac{\int_{\lambda_{min}}^{\lambda_{max}} I(\lambda) \cdot sin\left(\frac{2\pi(\lambda-\lambda_{min})}{(\lambda_{max}-\lambda_{min})}\right) d\lambda}{\int_{\lambda_{min}}^{\lambda_{max}} I(\lambda)\, d\lambda} \tag{3}$$

The components G and S are normalized to the entire integrated spectrum, where $\int_{\lambda_{min}}^{\lambda_{max}} I(\lambda)\, d\lambda$ corresponds to the toal intensity over the full spectral range. A polar diagram is appropriate for visualization of the phasor points. Thus, a vector with an angle φ and modulation M can be calculated:

$$\varphi = \tan^{-1}\left(\frac{S}{G}\right) \tag{4}$$

$$M = \sqrt{S^2 + G^2} \tag{5}$$

In Figure 1b (main manuscript) two exemplary spectra and their representation in a phasor plot are shown. A full circle represents the whole analyzed spectral range. Therefore, the angle reflects the wavelength of the peak of the spectrum. The modulation, on the other hand, corresponds to the broadness of the spectrum. The broader the spectrum, the closer to the center lies the phasor point.

For generating the spectral phasor directly with hardware components, the pixelwise spectrum $I(x, y, \lambda)$ is weighted with a sine and cosine transmission filter for a desired spectral range (λ_{min}, λ_{max}) resulting in an intensity at each pixel of:[2]

$$I_{sin}(x, y) = \int_{\lambda_{min}}^{\lambda_{max}} I(x, y, \lambda) \cdot sin\left(2\pi \frac{\lambda-\lambda_{min}}{\lambda_{max}-\lambda_{min}}\right) d\lambda \ \text{(analogous for } I_{\cos}\text{)} \tag{6}$$

Since the filters have a transmission range from 0 to 1, the pixel values need to be shifted to the interval from -1 to +1 to obtain G and S within the unity circle:[2]

$$G(x, y) = 2\,\frac{I_{\cos}(x, y)}{I_{total}(x, y)} - 1 \tag{7}$$

$$S(x, y) = 2\,\frac{I_{\sin}(x, y)}{I_{total}(x, y)} - 1 \tag{8}$$

2. Theoretical background of the polarization filtering to create a cosine transmission

To generate a cosine shaped transmission, a variable retarder is placed between two linear polarizers. Based on the Jones calculus, the transformation of the polarization state can be calculated. For a linear polarizer operating at an angle β the Jones matrix is:[3]

$$M_P = \begin{bmatrix} \cos^2(\beta) & \frac{1}{2}\sin(2\beta) \\ \frac{1}{2}\sin(2\beta) & \sin^2(\beta) \end{bmatrix} \tag{9}$$

and the matrix for a variable retarder is given by:[3]

$$M_R = \begin{bmatrix} 1 & 0 \\ 0 & e^{i\varphi} \end{bmatrix} \tag{10}$$

where φ is the set phase retardation of the variable retarder, which is given by $\varphi = 2\pi R/\lambda$ and depends on the wavelength λ and the retardance R.

When the incoming light is randomly polarized, the light has a linear polarization of angle α after the first linear polarizer:[3]

$$E_{in} = \begin{bmatrix} \cos(\alpha) \\ \sin(\alpha) \end{bmatrix} \tag{11}$$

This linear polarized light is passed through the variable retarder and the second polarizer, which acts like a filter for polarization. At the end, the output polarization vector E_{out} can be calculated as follows:

$$E_{out} = M_P \cdot M_R \cdot E_{in} = \begin{bmatrix} \cos^2(\beta) & \frac{1}{2}\sin(2\beta) \\ \frac{1}{2}\sin(2\beta) & \sin^2(\beta) \end{bmatrix} \cdot \begin{bmatrix} 1 & 0 \\ 0 & e^{i\varphi} \end{bmatrix} \cdot \begin{bmatrix} \cos(\alpha) \\ \sin(\alpha) \end{bmatrix} \tag{12}$$

For operating both linear polarizers at angle $\alpha = \beta = 45°$, the equation can be simplified to:

$$E_{out} = \begin{bmatrix} 1/2 & 1/2 \\ 1/2 & 1/2 \end{bmatrix} \cdot \begin{bmatrix} 1 & 0 \\ 0 & e^{i\varphi} \end{bmatrix} \cdot \begin{bmatrix} \sqrt{2}/2 \\ \sqrt{2}/2 \end{bmatrix} = \frac{\sqrt{2}}{4} \cdot \begin{bmatrix} 1 & 1 \\ 1 & 1 \end{bmatrix} \cdot \begin{bmatrix} 1 & 0 \\ 0 & e^{i\varphi} \end{bmatrix} \cdot \begin{bmatrix} 1 \\ 1 \end{bmatrix} \tag{13}$$

which gives a final output vector for the polarization of:

$$E_{out} = \begin{bmatrix} E_{0x} \\ E_{0y} \end{bmatrix} = \frac{\sqrt{2}}{4} \begin{bmatrix} 1 + e^{i\varphi} \\ 1 + e^{i\varphi} \end{bmatrix} \tag{14}$$

To get the intensity, the magnitude of the vector, which is given by multiplication of the complex conjugated form, is squared:

$$\begin{aligned} I = I_0(E_x E_x^* + E_y E_y^*) &= I_0 \left(\frac{\sqrt{2}}{4}\right)^2 [(1 + e^{i\varphi})(1 + e^{-i\varphi}) + (1 + e^{i\varphi})(1 + e^{-i\varphi})] \\ = I_0 \frac{1}{8}[4 + 2e^{i\varphi} + 2e^{-i\varphi}] &= I_0 \frac{1}{8}[4 + 2(\cos(\varphi) + i\sin(\varphi)) + 2(\cos(\varphi) - i\sin(\varphi))] \\ &= I_0 \frac{1}{2}[1 + \cos(\varphi)] \end{aligned} \tag{15}$$

The transmission through all polarizing elements can be written as:

$$T(\varphi) = \frac{I}{I_0} = \frac{1}{2}[1 + \cos(\varphi)] \tag{16}$$

with only a dependence on the phase retardation of the variable retarder $\varphi = 2\pi R/\lambda$.

By adjusting the term $R = d\Delta n(\lambda)$, where d represents the thickness of the retarder and $\Delta n(\lambda)$ is the birefringence of the retarder material, the cosine function can be spectrally tuned and shifted. Therefore, for a given wavelength range, the cosine-shaped transmission can be easily switched to a sine-shaped transmission. If the term $R = d\Delta n(\lambda)$ is set to 0 (or near zero), the transmission is independent on the wavelength, this position can be used as normalization for the spectral phasor. In a liquid crystal retarder, the birefringence of the liquid crystal itself shows

an additional wavelength-dependency, which slightly changes the transmission behavior. A good assumption for the dispersion of the birefringence are the first two terms of the Cauchy equation:[4,5]

$$\Delta n(\lambda) = A + \frac{B}{\lambda^2} \tag{17}$$

where A and B are intrinsic parameters of the liquid crystal material. Equation (16) can therefore be rewritten as:

$$T(\lambda) = \frac{1}{2}\left[1 + \cos\left(\frac{2\pi d\left(A + \frac{B}{\lambda^2}\right)}{\lambda}\right)\right] = \frac{1}{2}\left[1 + \cos\left(2\pi d\left(\frac{A}{\lambda} + \frac{B}{\lambda^3}\right)\right)\right] \tag{18}$$

with a thickness of the LC cell of d = 50 µm. Based on Equation (18) we fitted values of A = 0.19 and B = 0.013 µm² to the measured transmission curve when no voltage was applied to the LCVR (0 V). By applying a voltage, the effective birefringence of the LC cell decreases due to tilting of the liquid crystal molecules. This can be described by a voltage transfer function $g(V)$.[5] We obtained this function in the range from 0.5 to 3.0 V by fitting the measured transmission curves to the following equation:

$$T(\lambda) = \frac{1}{2}\left[1 + \cos\left(2\pi d\, g(V)\left(\frac{A}{\lambda} + \frac{B}{\lambda^3}\right)\right)\right] \tag{19}$$

where $g(0) = 1$ and higher voltages decreased this value. The retardance of the LCVR in dependence of the voltage and wavelength is therefore given by:

$$R(\lambda, V) = d\, g(V)\left(A + \frac{B}{\lambda^2}\right) \tag{20}$$

Consequently, the retardance slightly differs in the covered spectral range (900 to 1600 nm, see Figure S3b). Overall, this did not significantly affect our method for generating a cosine and sine transmission function and therefore we approximated the dispersion with B=0.

As we wanted to achieve the best fit to a perfect sine and cosine transmission, we also considered the negated sine and cosine modes. For this, the Equation (6) was adjusted:

$$I_{sin}(x, y) = \int_{\lambda_{min}}^{\lambda_{max}} I(x, y, \lambda) \cdot q_{sin} \cdot T_{sin}(\lambda)\, d\lambda \tag{21}$$

$$I_{cos}(x, y) = \int_{\lambda_{min}}^{\lambda_{max}} I(x, y, \lambda) \cdot q_{cos} \cdot T_{cos}(\lambda)\, d\lambda \tag{22}$$

The coefficients $q_{sin}$and q_{cos} can either be +1 or -1 depending on whether the best fit is a positive or negative sine and cosine function in a specific wavelength range. By applying the same coefficient to the G and S component, we would display the points in the phasor plot in a unit circle with start and end at G = 1, S = 0. The correction is given by the following equations:

$$G(x, y) = q_{cos}\, (2\frac{I_{\cos}(x, y)}{I_{total}(x, y)} - 1) \tag{23}$$

$$S(x, y) = q_{sin}\, (2\frac{I_{sin}(x, y)}{I_{total}(x, y)} - 1) \tag{24}$$

Another minor correction was used, due to reason that the used LCVR was not capable of reaching R = 0. Instead, a residual retardance of R =107 nm (at maximum voltage of 25 V, see Figure S2) resulted in an approx. 6 % lower overall transmission. Since this error was almost constant over the used spectral range (900 to 1600 nm), we applied a correction factor of 1.06 to the $I_{total}(x, y)$ image.

3. Phasor discrimination and spectral resolution

Theoretically, the discrimination or spectral resolution of two spectra in the phasor space improves with each zoom-in. However, there is a practical limit. Spectra have a finite linewidth at a certain phasor range. Thus, a further increase will not enhance the discrimination/resolution. The discrimination of two phasor points can be described by the Euclidean distance d. Moreover, the discrimination power depends on the spreading (noise) of the phasor points. The simplest form of a phasor point cluster can be described by an isotropic Gaussian distribution (Figure S2c). For simplification we transferred the problem in 1D space. The histogram along the line through both cluster's mean positions were plotted (Figure S2c,d). To distinguish these two Gaussians, a common definition is to normalize the distance of the peaks to the average of the two widths.[6] We used the average of full width half maxima $\frac{(w_1+w_2)}{2}$ as normalization, where a value of ≤ 1 indicate two unresolvable phasor clusters. Hence, we defined this condition as the phasor discrimination power (PDP), which can be calculated as follows:

$$PDP = \frac{d}{\frac{(w_1 + w_2)}{2}} = \frac{2d}{(w_1 + w_2)} \tag{25}$$

The distance d is given by the Euclidean distance and the FWHM w is connected to the standard deviation by $w = 2\sqrt{2\ ln2}\ \sigma$, the equation can be rewritten as:

$$PDP = \frac{\sqrt{(G_1 - G_2)^2 + (S_1 - S_2)^2}}{\sqrt{2\ ln2}\ (\sigma_{phasor1} + \sigma_{phasor2})} \tag{26}$$

where the mean phasor point (G_1, S_1) and $\sigma_{phasor1}$ standard deviation of the two input spectra are considered. The standard deviation or uncertainty σ_{phasor} of a phasor point is related to the noise of the input intensities $I_{cos}, I_{sin}, I_{total}$ for the G and S calculation (Equations (7) and (8)). To calculate the overall noise of a phasor point σ_{phasor} we need to take all noise source of the individual intensities $I_{cos}, I_{sin}, I_{total}$ into account:

$$\sigma_{cos} = \frac{I_{cos}}{10^{SNR/20dB}}, \quad \sigma_{sin} = \frac{I_{sin}}{10^{SNR/20dB}}, \quad \sigma_{total} = \frac{I_{total}}{10^{SNR/20dB}} \tag{27}$$

where SNR is the signal-to-noise ratio. To get the noise for the G and S component of the phasor one can use can use the propagation of uncertainty for the standard deviations:

$$\sigma_{G/S}^2 = \left(\frac{\partial G/S}{\partial I_{cos/sin}} \cdot \sigma_{cos/sin}\right)^2 + \left(\frac{\partial G/S}{\partial I_{total}} \cdot \sigma_{total}\right)^2 = \frac{8\left(\frac{I_{cos/cos}}{SNR}\right)^2}{{I_{total}}^2} \tag{28}$$

The overall standard deviation σ_{phasor} for a phasor point (G, S) is given by:

$$\sigma_{phasor} = \sqrt{\sigma_G^2 + \sigma_S^2} \tag{29}$$

This leads to the following equation for the standard deviation of the phasor:

$$\sigma_{phasor} = \frac{2\sqrt{2}}{10^{SNR/20dB}} \cdot \frac{\sqrt{{I_{cos}}^2 + {I_{sin}}^2}}{I_{total}} \tag{30}$$

Both phasor points have a standard deviation σ_{phasor} (same SNR for both spectra), therefore we can rewrite Equation (26) as:

$$PDP = \frac{10^{SNR/20dB}\sqrt{(G_1 - G_2)^2 + (S_1 - S_2)^2}}{4\sqrt{ln2}\left(\frac{\sqrt{{I_{cos1}}^2 + {I_{sin1}}^2}}{I_{total1}} + \frac{\sqrt{{I_{cos2}}^2 + {I_{sin2}}^2}}{I_{total2}}\right)} \tag{31}$$

We calculated the PDP of two spectra in a defined phasor range following these steps:

1. Generate two Gaussian spectra $I(\lambda)$ with the same FWHM at the peak wavelengths $\lambda_1 = 1250\,nm$ (middle wavelength of our spectral coverage from 900 to 1600 nm) and $\lambda_2 = \lambda_1 + \Delta\lambda$ (Figure S2a)
2. Define the phasor range with λ_1 being the middle: $\lambda_{min} = \lambda_1 - \frac{(\lambda_{max}-\lambda_{min})}{2}$ and $\lambda_{max} = \lambda_1 + \frac{(\lambda_{max}-\lambda_{min})}{2}$
3. Generate transmission filter curves $T_{sin}(\lambda, R_{sin})$ and $T_{cos}(\lambda, R_{cos})$ for this specific phasor range (λ_{min} to λ_{max}) following Equation (16) and fit to a perfect sine and cosine wave for this phasor range (Figure S2b)
4. Calculate the cosine, sine and total intensity fractions: $I_{cos1}, I_{sin1}, I_{total1}$ and $I_{cos2}, I_{sin2}, I_{total2}$
5. Spectra are transformed into the phasor space: (G_1, S_1) and (G_2, S_2)
6. PDP is calculated by taking a defined SNR into account

Based on this calculation, the PDP was plotted against the phasor range (10 to 700 nm). We varied the peak distance $\Delta\lambda$ between the Gaussian spectra (1 nm, 5 nm, 20 nm) and adjusted the FWHM of both spectra subsequently (from 10 to 100 nm). Furthermore, we compared these calculations for two different signal-to-noise ratios (30 dB, 50 dB). For narrow spectra, PDP improved with smaller phasor ranges, but there was a limit beyond which narrower phasor ranges decreased the PDP (Figure S2). This limit depended on the FWHM of the spectrum. For broad peaks (FWHM > 30 nm), the PDP was poor both at small and large phasor ranges. If the peak wavelength was increased by a factor of 5x or 20x, the overall PDP was improved by the same factor. Consequently, a decrease in SNR by 20 dB resulted in a tenfold reduction in PDP.

4. Spectral unmixing of NIR fluorophores

If a spectrum consists of a mixture of multiple known spectra, this can be used to unmix the concentration of these spectral components. The detected spectrum in each pixel (x,y) is a linear combination of the single spectra of n components:

$$I_n(x,y) = \sum_{k=m}^{n} a_m\, I_m \tag{32}$$

The value a_m represents the fraction of the spectral component I_m inside a pixel. This linear combination also remains valid after the Fourier transformation in the phasor space[1,2]. If there is a mixture of the components $I_1(\lambda)$ and $I_2(\lambda)$, the individual values for G are given by:

$$G_1 = \frac{\int_{\lambda_{min}}^{\lambda_{max}} I_1(\lambda)\, \cdot\, T_{cos}(\lambda)\, d\lambda}{\int_{\lambda_{min}}^{\lambda_{max}} I(\lambda)\, d\lambda} \tag{33}$$

$$G_2 = \frac{\int_{\lambda_{min}}^{\lambda_{max}} I_2(\lambda)\, \cdot\, T_{cos}(\lambda)\, d\lambda}{\int_{\lambda_{min}}^{\lambda_{max}} I(\lambda)\, d\lambda} \tag{34}$$

where $I(\lambda)$ is the normalization over the sum of both spectral components. For the linear combination thereof, we get:

$$G_{1,2} = \frac{\int_{\lambda_{min}}^{\lambda_{max}} a_1\, I_1(\lambda)\, \cdot\, T_{cos}(\lambda)\, d\lambda}{\int_{\lambda_{min}}^{\lambda_{max}} I(\lambda)\, d\lambda} + \frac{\int_{\lambda_{min}}^{\lambda_{max}} a_2\, I_2(\lambda)\, \cdot\, T_{cos}(\lambda)\, d\lambda}{\int_{\lambda_{min}}^{\lambda_{max}} I(\lambda)\, d\lambda} = a_1 G_1 + a_2\, G_2 \tag{35}$$

The components are mixed in fractions of a_1 and a_2, which together are normalized to $a_1 + a_2 = 1$. This simplifies the Equation (35) to:

$$G_{1,2} = a_1 G_1 + (1 - a_1) G_2 \tag{36}$$

If we know the pure G_1 and G_2 values of our sample, we can measure $G_{1,2}$ and unmix the concentrations of the components:

$$a_1 = \frac{G_{1,2} - G_2}{G_1 - G_2} \tag{37}$$

The phasor point for a mixed spectrum of two components is always lying on the connecting line between the individual phasor points. This calculation is analogous for the S values.

5. Correction for inhomogeneity in macroscopic images

For macroscopic images the G and S components were corrected by a correction matrix based on a measurement with a white reflectance standard. $I_{cos,corr}(x,y)$, $I_{sin,corr}(x,y)$ and $I_{total,corr}(x,y)$ represent the normalized intensity of the white reflectance standard for the three images. By division of these matrices the images were homogenized, which corrects the uneven illumination and the spatial inhomogeneity of the LCVR. Based on Equation (16) we get:

$$G(x,y) = q_{cos}\ (2\frac{I_{\cos}(x,y)/I_{cos,corr}(x,y)}{I_{total}(x,y)/I_{total,corr}(x,y)} - 1) \tag{38}$$

$$S(x,y) = q_{sin}\ (2\frac{I_{\sin}(x,y)/I_{sin,corr}(x,y)}{I_{total}(x,y)/I_{total,corr}(x,y)} - 1) \tag{39}$$

To avoid repetitive calculations, we defined a correction matrix for G and S:

$$G_{corr} = \frac{I_{total,corr}(x,y)}{I_{cos,corr}(x,y)} \tag{40}$$

$$S_{corr} = \frac{I_{total,corr}(x,y)}{I_{sin,corr}(x,y)} \tag{41}$$

Equations (38) and (39) can therefore be simplified as:

$$G(x,y) = q_{cos}\left(2\ G_{corr}\frac{I_{\cos}(x,y)}{I_{int}(x,y)} - 1\right) \tag{42}$$

$$S(x,y) = q_{sin}\left(2\ S_{corr}\frac{I_{sin}(x,y)}{I_{int}(x,y)} - 1\right) \tag{43}$$

Supplementary Videos

Video S1. HyperNIR imaging of water uptake in a leaf. *The video shows the visible, NIR and HyperNIR image of a leaf from a pepper plant as well as the calculated phasor plots for a duration of 2.5 h. After 1 h of the measurement the plant was watered and water uptake started. The Figure S12 and Figure 5b (main manuscript) show measurement at selected time points of this video. Note that the normalized rate of change is the relative change in the Euclidean distance in phasor space for each pixel. Scale bar = 1 mm.*

Supplementary Figures and Tables

Table S1: Commercially available hyperspectral snapshot cameras.

	Resolution				
**Company**	**Spectral (bands)**	**Spatial (pixel)**	**Temporal (Hz)**	**Range (nm)**	**Link**
Cubert	38	200x200	80	980 – 1650	https://cubert-hyperspectral.com/en/ultris-swir-1/
Bodkin	60	40x33	31.6	1000 – 1700	https://www.bodkindesign.com/products/swir-60/
imec	16	40x32	max. 120	1100 – 1650	https://www.imechyperspectral.com/en/real-time-hyperspectral-snapshot-cameras
SILIOS Technologies	9	432x344	68	1100 – 1700	https://www.silios.com/cicada-cam
Spectral Devices	16	135x105	100	1125 – 1640	https://spectraldevices.com/products/16-band-swir-camera

Table S2. Costs to implement HyperNIR imaging (without mechanical parts).

**High light conditions**		
Part	Pieces	Costs (€)
LPIREA100-C, Thorlabs	2	185.73
LCC1115-B, Thorlabs	1	826.07
KLC101, Thorlabs	1	798.30
		1,995.83
**Low light conditions (more efficient transmission components desirable)**		
Part	Pieces	Costs (€)
LPNIR100, Thorlabs	2	935.12
LCC1115-B, Thorlabs	1	826.07
KLC101, Thorlabs	1	798.30
		3,494.61

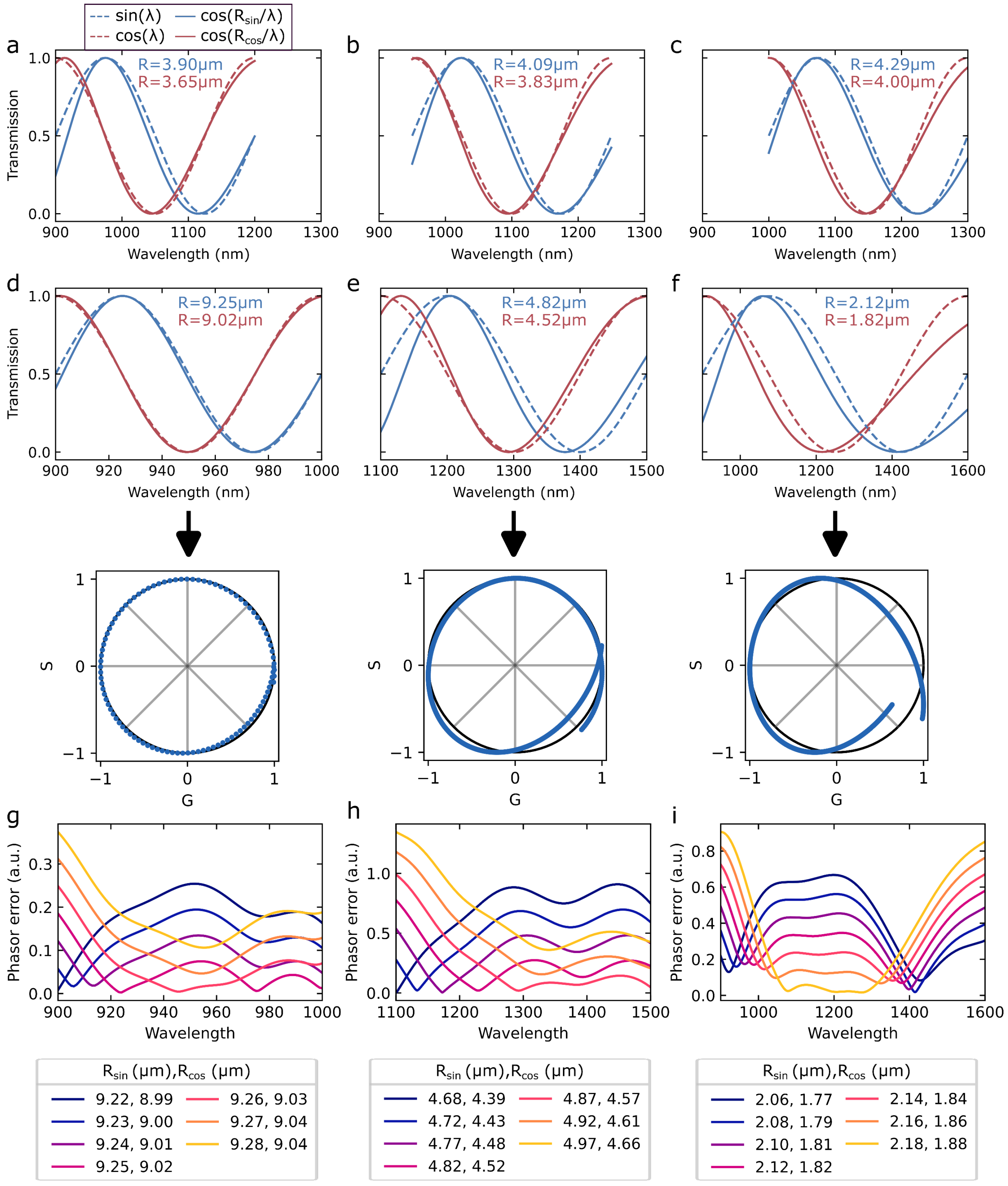

Figure S1. Comparison of theoretical (perfect) cos(λ)/sin(λ) versus cos(R/λ) functions. *a) – f) Transmission functions for different retardances R generated by a variable retarder, which allows the transmission to be precisely matched to a desired spectral range. The value for R was found by fitting the cos(R/λ) function to the perfect cos(λ)/sin(λ) (python package numpy.curve_fit). The bottom part in (d) – (f) shows the sine and cosine function mapped onto the phasor space for every wavelength of the phasor range. g) – i) Error in position of the phasor point in relation to a phasor point calculated with a perfect sine and cosine (Euclidean distance). To showcase the possibility to minimize this error for specific wavelengths, the retardance of* R_{sin} *and* R_{cos} *was adjusted. Note that the curves in pink color corresponds to the transmission curves in (d) – (f).*

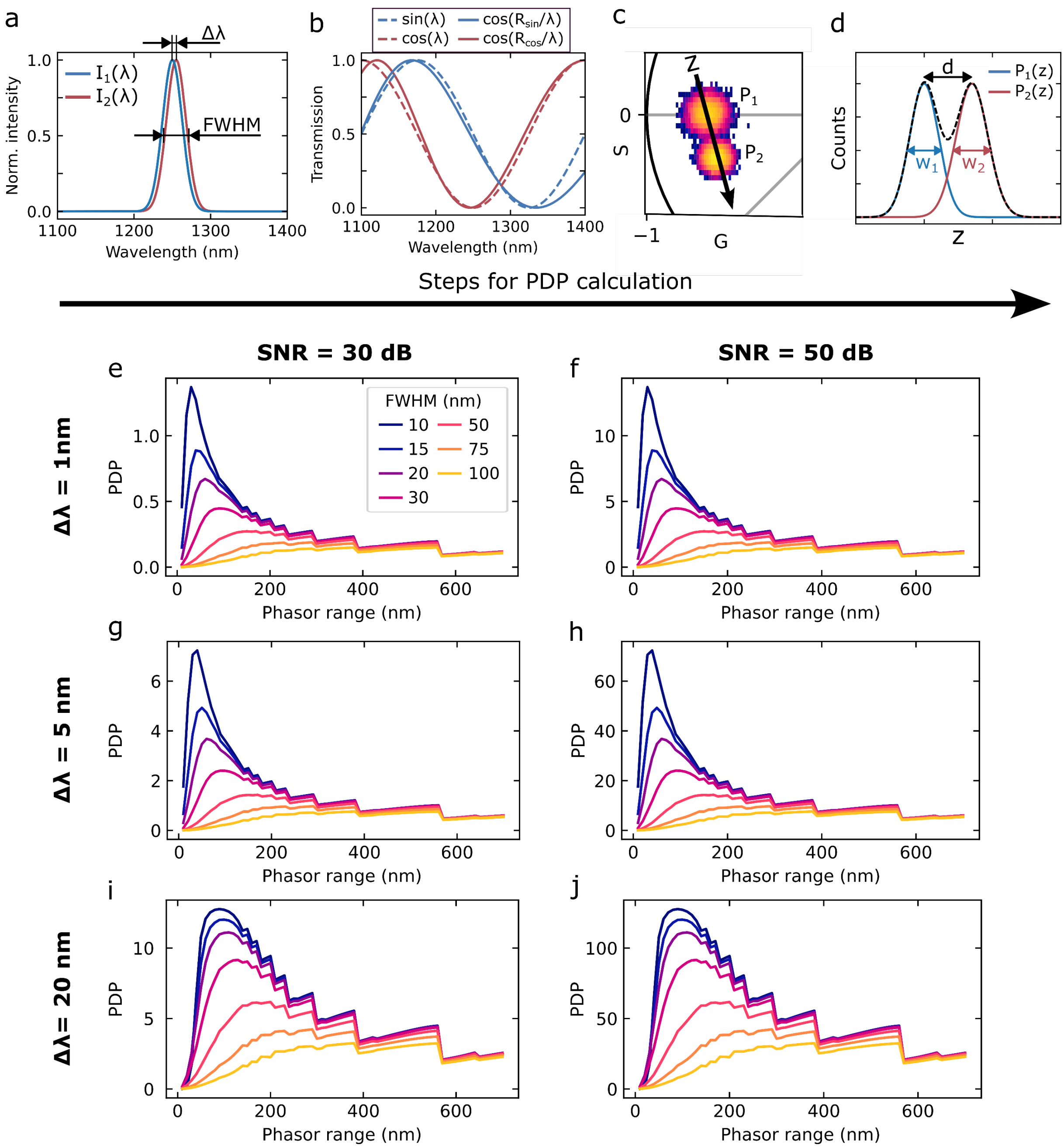

***Figure S2. Discrimination of two spectral features in the phasor plot with varying phasor range**. a-d) Steps for the Phasor Discrimination Power (PDP) calculation. a) Two Gaussian spectra with the same FWHM were assumed and a wavelength difference of their peak of Δλ. b) The spectra were transformed by a sine and cosine transmission filter for a defined phasor range (see Figure S1a-f). c) Phasor clusters of two spectra from (a) with an isotropic Gaussian distribution with standard deviation σ_{phasor} (dependent on signal-to-noise ratio (SNR)). d) Histogram along the line z from (c). Two Gaussian peaks with a full width half maximum of w_1 and w_2 and a distance of d. Based on d, w_1 and w_2 the PDP was calculated (details in Supplementary section 3). e-i) PDP in relation to the range of the phasor. e) Δλ = 1 nm & SNR = 30 dB, f) Δλ = 1 nm & SNR = 50 dB, g) Δλ = 5 nm & SNR = 30 dB, h) Δλ = 5 nm & SNR = 50 dB, i) Δλ = 20 nm & SNR = 30 dB, j) Δλ = 20 nm & SNR = 50 dB. Note that the saw-tooth-like shape is a computational artifact given the discrete estimation of the best transmission curves in (c).*

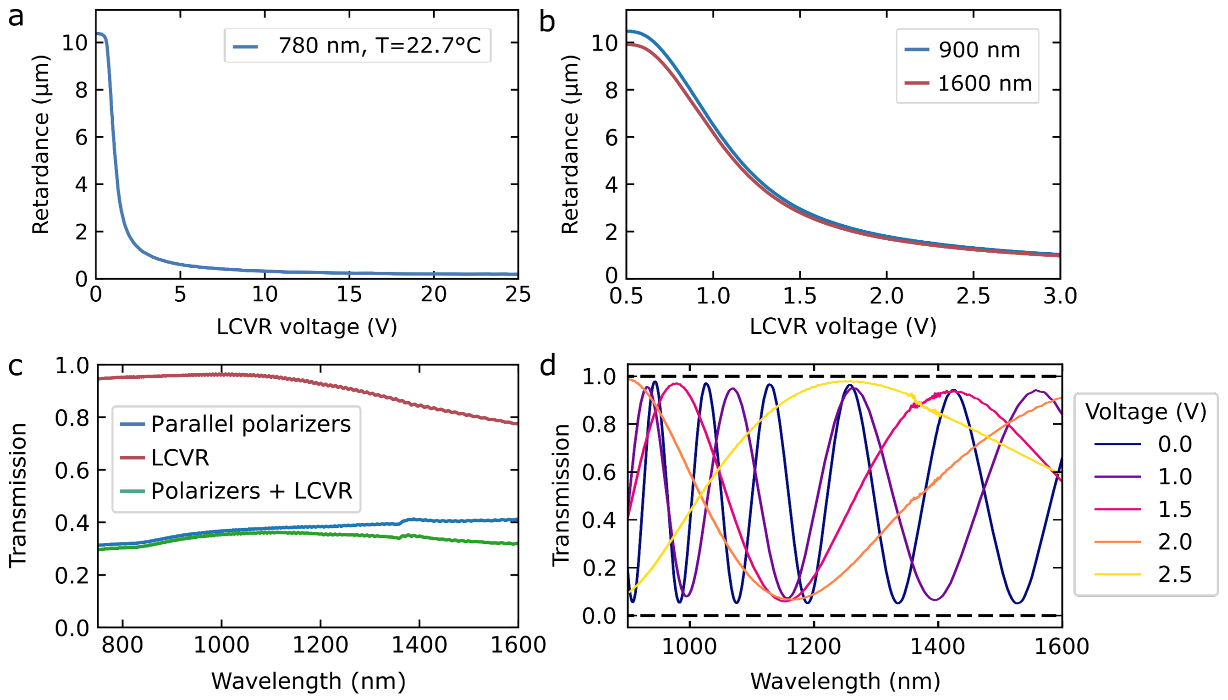

Figure S3. Voltage-adjustable retardance. *a) Working curve of the used liquid crystal variable retarder (LCVR), measured from the manufacturer (Thorlabs) at 780 nm and 22.7°C. By adjusting the LCVR input voltage the retardance can be tuned. b) The largest retardance change is found in the range between 0.5 V and 3.0 V. We obtained this curve by fitting Equation (19) to the measured transmission spectra. Increasing the wavelength results in a slight decrease in retardance, due to the dispersion of the birefringence of the liquid crystal material. Shown are the minimum (900 nm) and maximum (1600 nm) wavelength covered with HyperNIR c) Spectral transmission of the optical components with random polarized light as input. For the complete HyperNIR setup (Polarizers + LCVR) the minimum retardance was selected (R =107 nm at a voltage of 25 V). d) Transmission spectra of the HyperNIR setup for various voltages at the LCVR. The transmission was normalized to the transmission curve of the optical components from (c).*

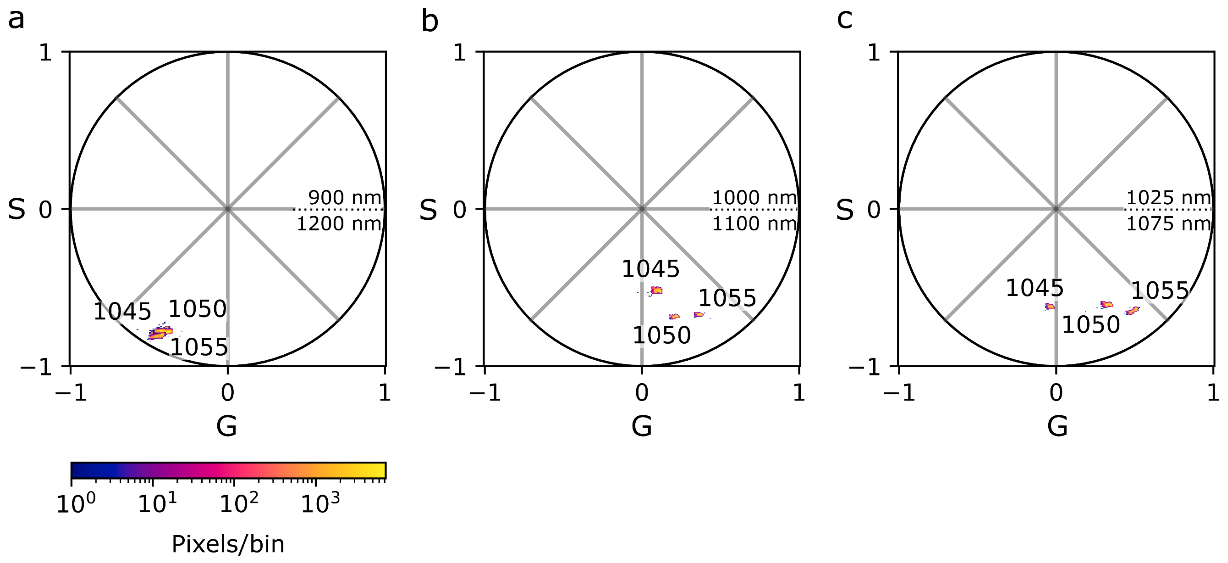

Figure S4. Spectral phasors of NIR light signals for different spectral ranges. *Differentiation of spectra with peaks at 1045 nm, 1050 nm and 1055 nm (monochromator with xenon-arc lamp, FWHM = 15 ± 2 nm) for different adjusted spectral ranges / spectral resolution. Ranges: a) 900 – 1200 nm, b) 1000 – 1100 nm, c) 1025 – 1075 nm.*

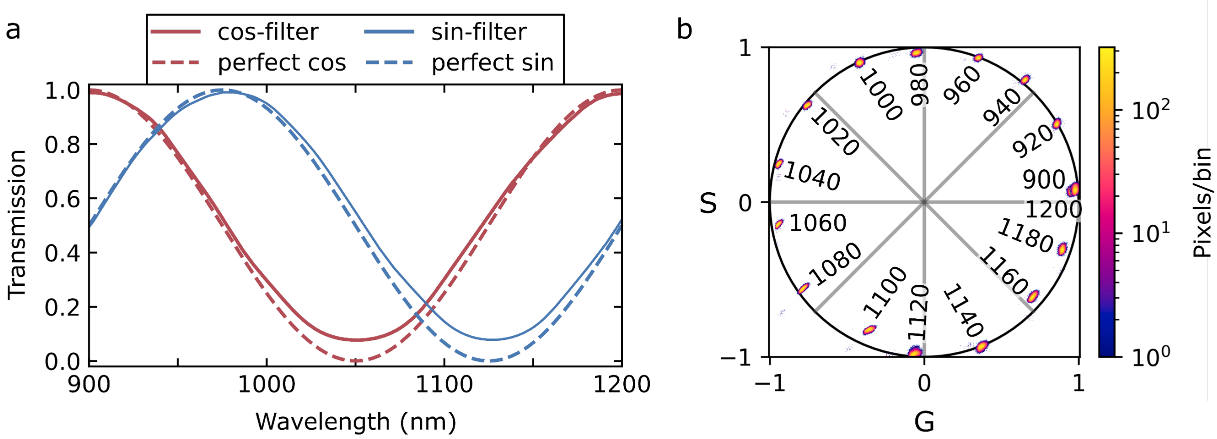

Figure S5. NIR phasors with hardware-based sine and cosine spectral filters. *a) Custom-made interference filters (OptoSigma, costs: 7,754 € for a filter set) designed for the spectral range 900 – 1200 nm to create a sine and cosine transmission. Note that a transmission minimum of 5 % was chosen, since 0 % transmission would lead to an image with noise as a main intensity source. b) Reference measurement with a monochromatic light source (monochromator with xenon-arc lamp, FWHM = 15 ± 2 nm).*

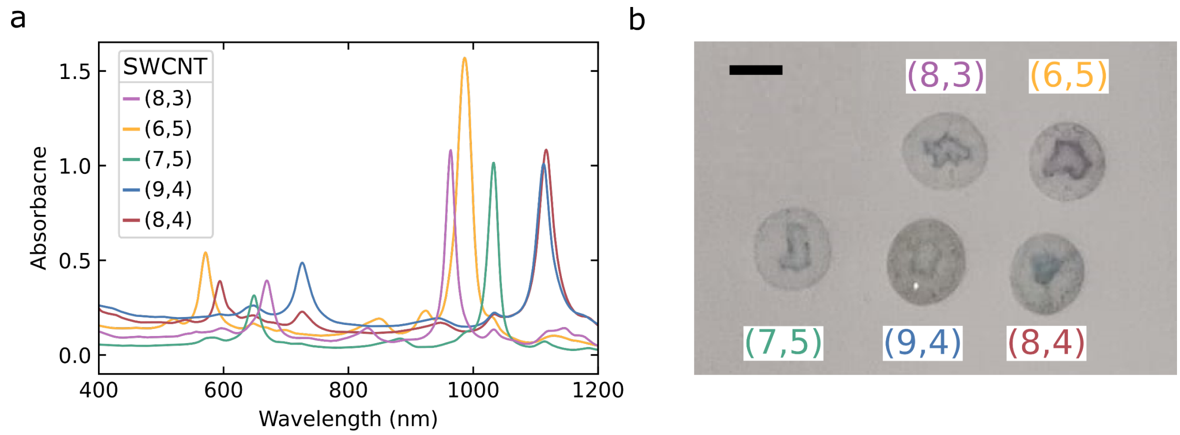

Figure S6. NIR fluorescent SWCNT samples. *a) Absorptions spectra of SWCNTs of different chirality. b) SWCNTs dropcasted on a PVDF membrane imaged with a smartphone camera. Scale bar = 1 mm.*

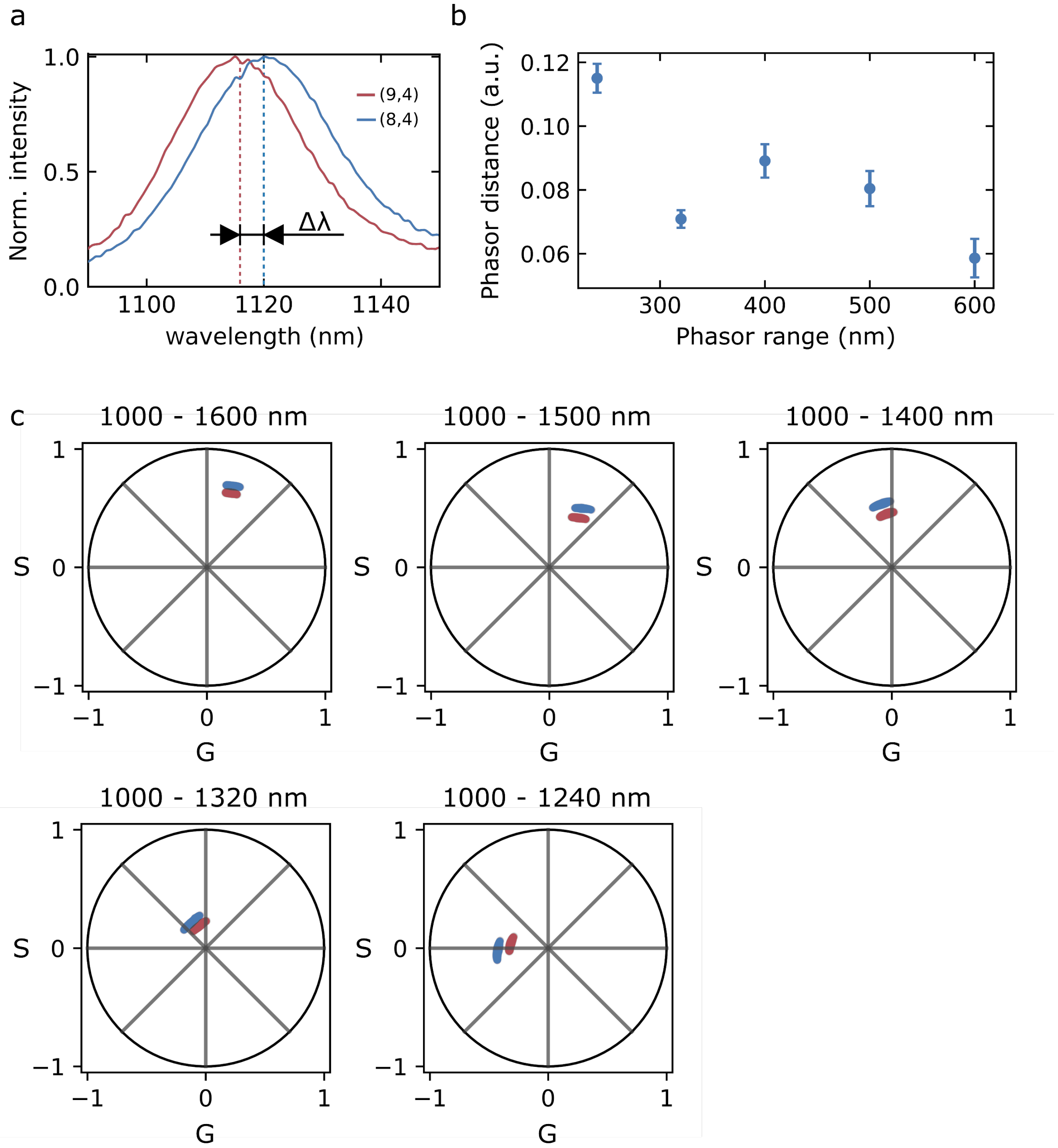

Figure S7. Spectral discrimination of similar NIR fluorophores and tuning of spectral resolution. *Differentiation of the (9,4)- and (8,4)-SWCNTs in a phasor plot. a) Emission spectra of the (9,4)- and (8,4)-SWCNTs. b) The calculated mean phasor distance (Euclidean distance) between the phasors of the (9,4)- and (8,4)-SWCNTs for different spectral ranges. Data = Mean ± SD, n = 3. c) Phasor plots of (9,4)- and (8,4)-SWCNTs for these different spectral ranges.*

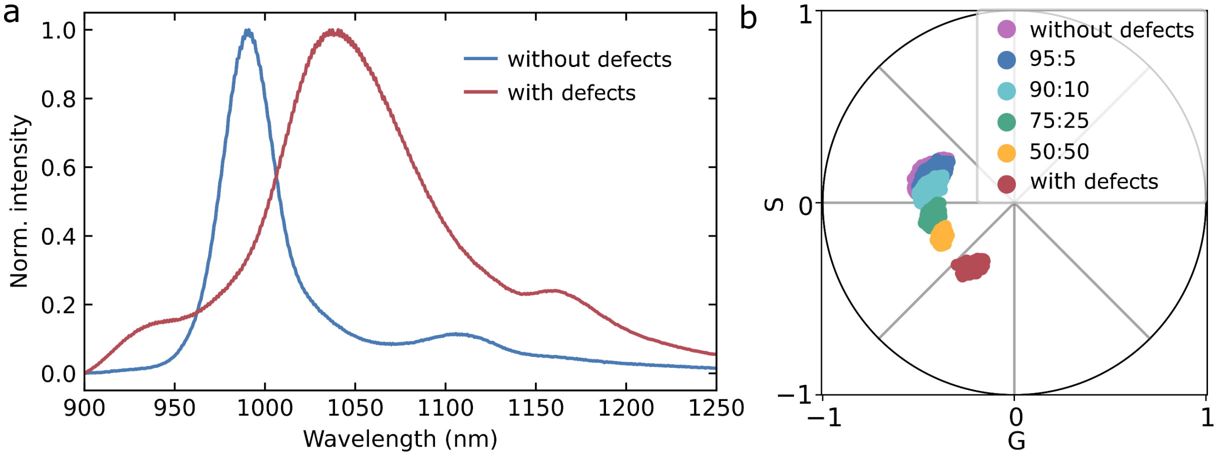

Figure S8. Spectral unmixing of SWCNTs with different emission wavelengths based on guanine quantum defects. *a) Normalized emission spectra of DNA modified SWCNTs with and without guanine quantum defects. Note that the emission peak position is shifted and the spectrum is broadened when increasing the ratio of SWCNTs with guanine quantum defects. b) Phasor plot for different mixtures of SWCNTs with and without defects in ratios with increasing amounts of SWCNTs without defects. The phasor position can be used to determine the concentration by unmixing.*

Table S3. Spectral unmixing of mixtures with SWCNTs with and without guanine quantum defects. *Data = Mean ± SD, n = 1500 (pixels).*

**Mixture**		**Experimentally determined**	
Without defects	With defects	Without defects	With defects
50 %	50 %	50 ± 3 %	50 ± 3 %
75 %	25 %	71 ± 3 %	29 ± 3 %
90 %	10 %	90 ± 2 %	10 ± 2 %
95 %	5 %	96 ± 2 %	4 ± 2 %

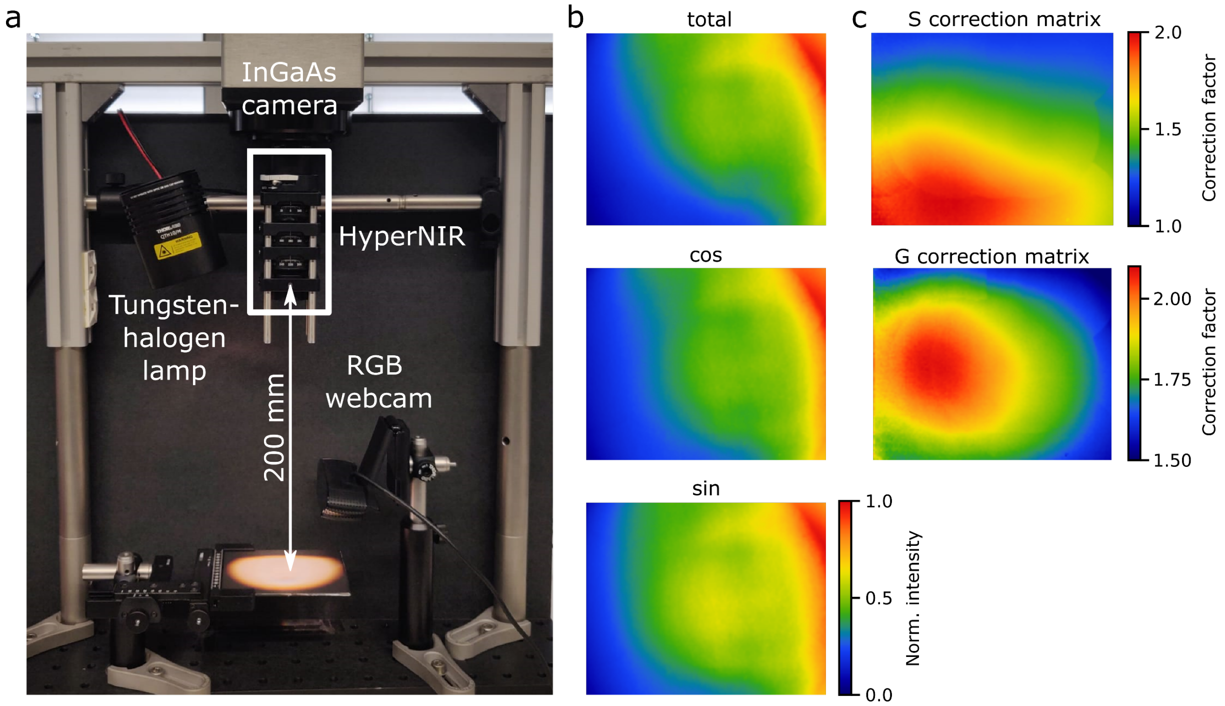

Figure S9. Macroscopic reflectance imaging. *a) Optical setup. b) Intensity images of a white reflectance standard for the phasor range 900 to 1600 nm. c) Calibration by correction matrices for the G and S component, which are computed by* S_{corr} *and* G_{corr} *(see Supplementary section 5, Equations (42) & (43)).*

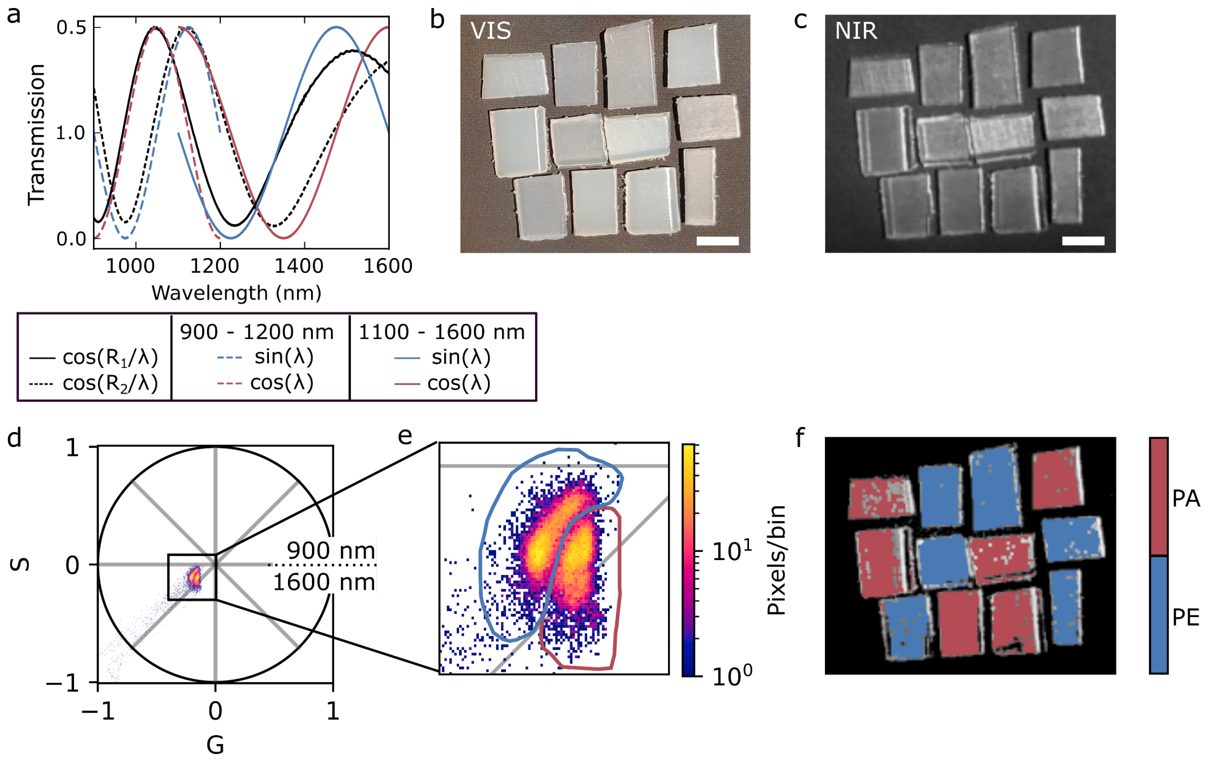

Figure S10. Multi-order phasor for plastic differentiation. *a) Transmission curve for two different retardances (black). Due to the multi-order cosine wave two different perfect sine and cosine functions are represented (ranges: 900 to 1200 nm and 1100 to 1600 nm). b) Visible image of pieces of PA and PE. c) NIR intensity image. d) Corresponding HyperNIR phasor plot for the range 900 to 1600 nm. e) Magnified plot of the phasor points in (d). f) HyperNIR image (dual-color coding based on the pixel position in the phasor plot according to the blue and red circles in (e). Scale bar = 0.5 mm.*

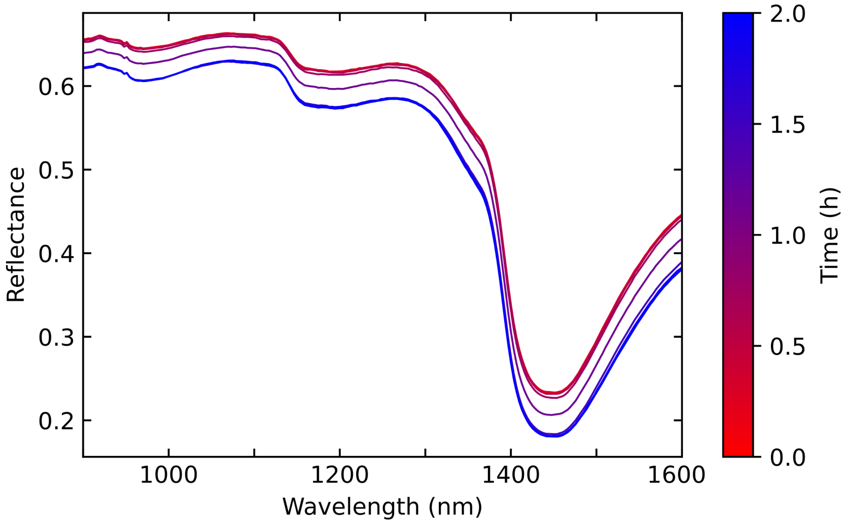

Figure S11. NIR spectral variation during water uptake. *The spectral reflectance of a region of interest (focus at the middle of the plant) of a leaf from the pepper plant was measured with an InGaAs spectrometer (AvaSpec-NIR256-1.7-HSC-EVO, Avantes) over 2 h. During the measurement the plant was watered (see Methods section).*

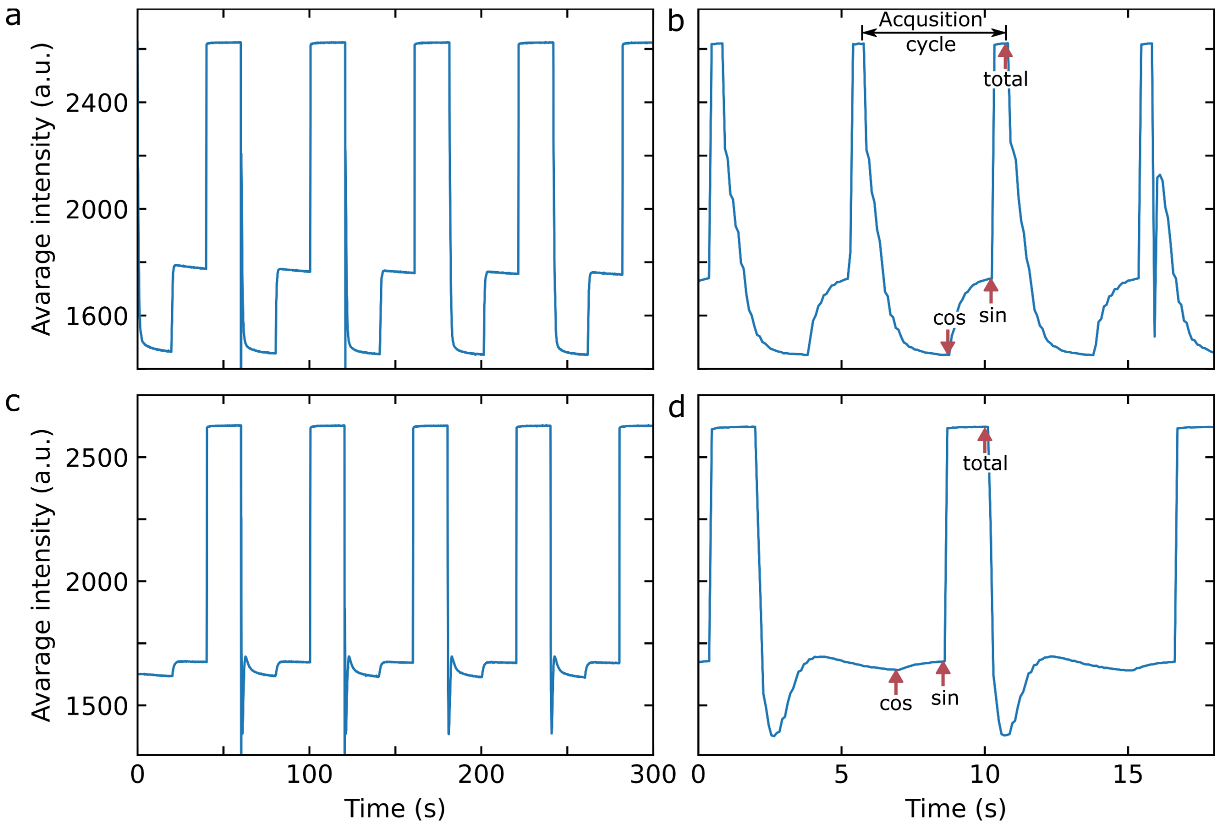

Figure S12. Switching time of the LCVR limits video rate. *a) The average in the image during switching of the LCVR driving voltage in the cycle 1.94 V, 2.19 V & 25 V. The time after the next voltage was applied was 10 s. b) Based on the plot in (a) the minimal time until the intensity was steady, was measured and applied as a cycle time for a HyperNIR video. For these voltages the cycle is: set 1.94 V → pause 1.5 s → set 2.19 V → pause 0.5 s → set 25 V → pause 3 s → set 1.94 V. c) The average intensity for a different voltage cycle: 1.35 V, 1.445 V & 25 V. d) Based on (c) the minimal times for the cycle were: set 1.35 V → pause 1.5 s → set 1.445 V → pause 1.5 s → set 25 V → pause 5 s → set 1.35 V. The time points at which the three images (cos, sin, total) were taken are marked.*

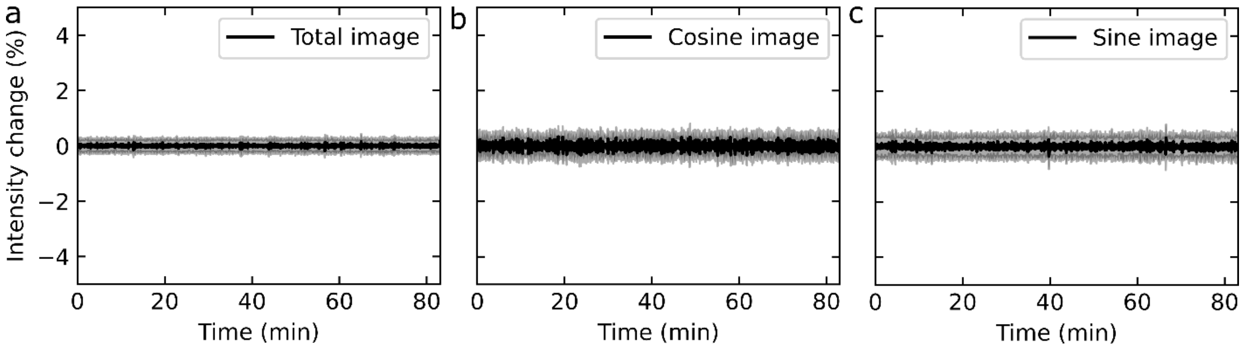

Figure S13. Intensity fluctuations I(t) of camera images between I_{t-1} and the next frame I_t, calculated as the intensity change = $\frac{I_{t-1}-I_t}{I_{t-1}} \cdot 100\%$. *We calculated the mean and SD of the intensity change over all pixel of the image (256x320) and plotted the results against the time. a) Total image, b) cosine image frame, c) sine image frame. Data = mean ± SD (n=81920). The shaded area represents the standard deviation.*

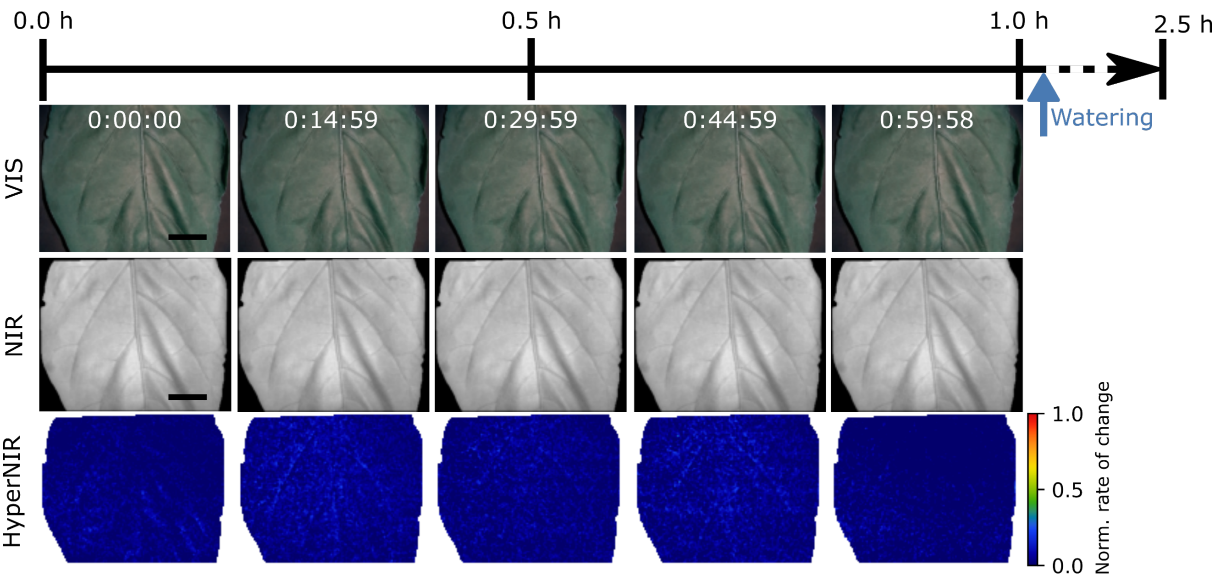

Figure S14. Dry leaf measured over a duration of 1 h. *Measurement of the time before the measurement for water uptake was started. The Figure 5b (main manuscript) shows the following 1.5 h of this measurement (see also Supplementary Video 1). Note that the normalized rate of change is the relative change in the Euclidean distance in phasor space for each pixel. Normalization of the rate of change was calculated similar to Figure 5b. Scale bar = 1 mm.*

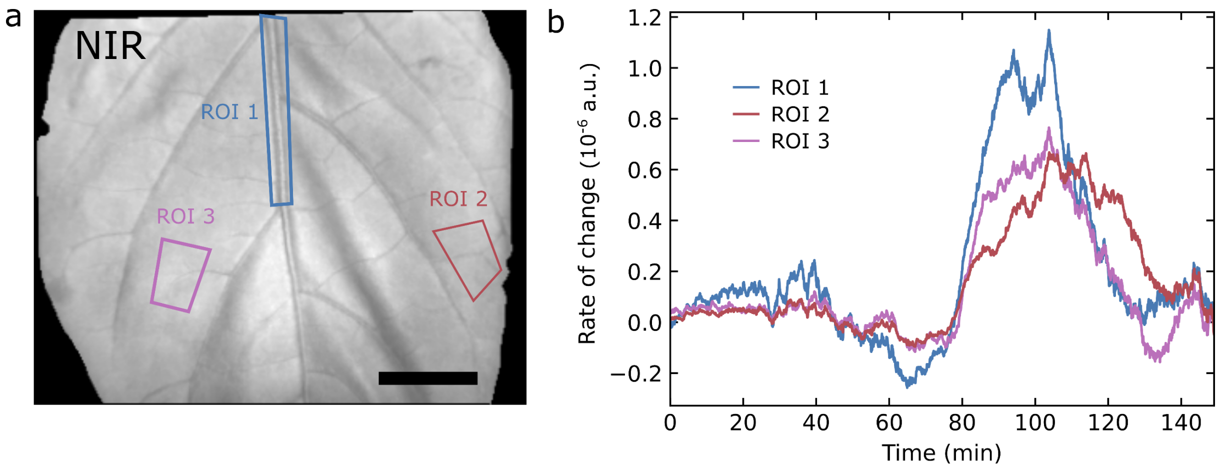

Figure S15. Phasor changes in response to water uptake for different regions of interests in a leaf. *a) Leaf with three regions of interests: ROI 1 is the main capillary of this leaf, ROI 2 and ROI 3 are different tissue parts, which represent the area around a small capillary. b) The mean rate of change in the different ROIs was calculated for every time frame of the video (Supplementary Video 1). Note that the rate of change is the relative change in the Euclidean distance in phasor space for each pixel. Scale bar = 1 mm.*

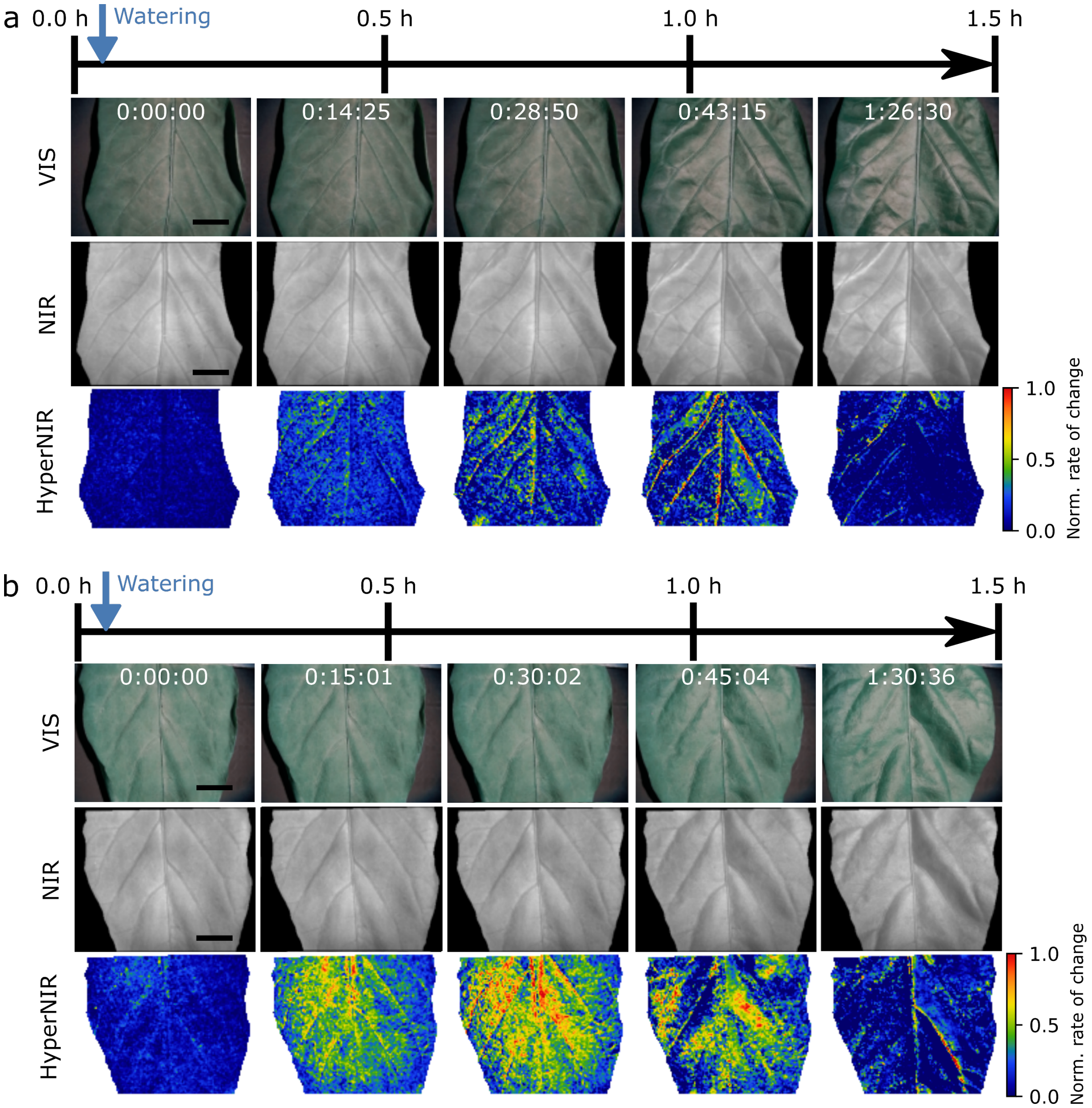

Figure S16. HyperNIR imaging of water uptake in leaves. *After approx. 1 min the plant was watered. In the HyperNIR images the normalized rate of change is visualized, which represents the movement of the phasor relative to the phasor position at the beginning for each pixel. a) and b) are different leaves. Note that the normalized rate of change is the relative change in the Euclidean distance in phasor space for each pixel. Scale bar = 1 mm.*

References

[1] F. Fereidouni, A. N. Bader, H. C. Gerritsen, *Opt Express* **2012**, *12*, 12729–12741.

[2] A. Dvornikov, E. Gratton, *Biomed Opt Express* **2018**, *8*, 3503–3511.

[3] E. Hecht, *Optics* 5 ed/fifth edition, global edition, Pearson. Boston **2017**

[4] J. Li, C.-H. Wen, S. Gauza, R. Lu, S.-T. Wu, *J. Display Technol.* **2005**, *1*, 51–61.

[5] A. Vargas, R. Donoso, M. Ramírez, J. Carrión, M. Del Mar Sánchez-López, I. Moreno, *OPT REV* **2013**, *5*, 378–384.

[6] P. Sandra, *J. High Resol. Chromatogr.* **1989**, *2*, 82–86.
